# Peristaltic Motion Enabled by Pneumatic Artificial Muscles (PAMs) as Structural “Soft–Stiff” Actuators in a Modular Worm-Inspired Robot

**DOI:** 10.3390/biomimetics9080447

**Published:** 2024-07-23

**Authors:** Beth Tinsley, Sergio Caponi, Lucy McAteer, Gleb Nebesnyy, Dean Sammanthan, Ella Sonia Keza, Parvez Alam

**Affiliations:** School of Engineering, The University of Edinburgh, Robert Stevenson Road, Edinburgh EH9 3FB, UK

**Keywords:** worm-inspired robot, biomimetic design, bioinspired robot, pneumatic artificial muscles, motion control, peristaltic motion

## Abstract

This paper considers the design, manufacture, and testing of a prototype “soft–stiff” worm-inspired robot referred to herein, as the **P**neumatically**A**ctuated Perista**L**tic **A**dvancing **M**odular (PALAM) robot. The robot has a modular structure, mimicking the segmented nature of earthworms, and each segment is individually actuated by a set of three pneumatic artificial muscles (PAMs). The PAMs contract when inflated by pressurised air, generating a pulling force and fulfilling the role of biological muscles in the robot. The PAMs are made from the elastomer silicone rubber, which affords the robot flexibility and enables a wide range of real-life applications. A control-system is designed which can inflate any PAM on demand, and hence replicate the peristaltic motion of earthworms in the PALAM robot. Finally, this paper discusses a successful, low-cost, and widely accessible approach for the manufacture of the PAMs utilised herein. The PAMs can be scaled dimensionally and made from different materials with varying mechanical properties and behaviours, meaning that they are suitable for use in a wide range of robotics applications.

## 1. Introduction

Soft-bodied invertebrates are present in almost every region of Earth and show great diversity in the spaces they inhabit [1]. The segmented worm family comprises around 16,500 species [1] and earthworms, the inspiration for this report, consist of over 7000 species alone [2]. This success can be greatly attributed to their robust locomotion capabilities and a segmented nature [3], which allow for a variety of movements and many degrees of mechanical freedom. The imitation of these characteristics in robotics leads to dynamic, flexible, and resilient designs. This presents myriad opportunities where high levels of adaptability are required, such as search and rescue applications, from navigating natural disaster zones to inaccessible caves. A locomotion technique notable for mimicry is ‘peristaltic motion’, which utilises different sets of contracting and elongating muscles.

From an engineering perspective, the locomotion capabilities displayed by soft-bodied invertebrates can be categorised into distinct motion techniques. Four of the most widely recognised are serpentine motion, concertina motion, caterpillar motion, and peristaltic motion [3,4]. Serpentine motion, or lateral undulation, is the side-to-side motion most often associated with snakes, while concertina motion involves snakes coiling into alternating curves before straightening and propelling themselves forward [3]. Both caterpillar and peristaltic motion are examples of rectilinear movement, demonstrating motion in a straight line in the direction of travel [5]. Caterpillar motion requires transverse undulating waves of muscle movement to progress forwards, and peristaltic motion relies on longitudinal waves of contractions and elongations moving from anterior to posterior along their bodies [6]. Earthworms achieve peristaltic motion using two different sets of muscles: circular muscles that loop around each segment, and longitudinal muscles that run along the length of the body [6]. Where the circular muscles contract, the earthworm is thinner and elongated, and where the longitudinal muscles contract, the earthworm is wider and contracted. To achieve peristaltic motion, the front of the body is anchored (with bristles, called ‘setae’) and contracted, pulling the rest of the body forward. Once the front setae are withdrawn, and the rear setae anchored, the circular muscles are used to lengthen the body, pushing it forwards again [6]. Compared with other locomotion techniques, peristalsis requires a minimal frontal area, making it the optimum mechanism for movement in narrow spaces (especially useful for mimicry in search and rescue robotics). Moreover, peristaltic motion enables worms to maintain a flat and stable posture relative to the ground, minimising risk of falling or tipping on uneven terrains.

Engineering efforts to utilise peristaltic movement have led to wide-ranging analyses and an improved understanding of the principles involved [1,3,7,8]. The serial homology (repeating sections) of segmented worms [1] are often modelled with modular systems, involving simplifications with lumped masses and connections in the form of actuators—from artificial muscles [3] to springs, dampers, [7,8] and idealised rigid rods. Additionally, the periodic movement of individual parts in peristalsis allows for the numerical analysis models of waves passing along a worm’s body during movement [9,10]. Friction has been proven crucial to peristaltic motion [3,11,12] as the maximum thrust exerted by the worm is dependent on the friction forces available to resist it [13]. The setae rely on friction for successful anchoring [3].

While there is a comparatively minimal development of worm-inspired robots with the specific aim of search and rescue, peristaltic motion has been extensively explored in the general robotics field. Robotic peristaltic motion employs the use of technologies such as vacuum pressure, pneumatic pressure, magnetic fluids, and shape-memory alloys [13,14,15,16]. Across all peristaltic models, however, the same movement principles apply. The actuation of individual sections must follow the specific order demonstrated by the earthworm [14]. Movement-centric design can allow worm-inspired robots to achieve a wider range of motion paths than the single straight line offered by simple peristaltic motion. Some techniques for optimisation include three-dimensional animation gesture studies, skeleton prototypes, video prototyping, and the use of interactive DoF software tools to complement 3D modelling [14,15,16]. Robotics can be classified by material into soft- and hard-bodied designs [17]. Traditionally, engineering has shown a preference for hard bodies, consisting of rigid members connected at discrete joints [18]. However, in recent years, the field of soft-bodied robotics, utilising compliant materials, has received increasing attention [19]. In worm-inspired robotics, there has been a notable development of hard-bodied designs, ranging from the use of planar link chains [20] to individually 3D-printed cells and the use of CNC machining [10]. However, soft-bodied designs lend themselves to the imitation of deformable natural systems [6], and as such, have seen comparatively more development in worm-inspired robotics [19]. The benefits of soft-bodied robots are significant. Their compliant and often more lightweight designs aid safe interactions with the environment and human users. Their flexibility also lends itself to following complicated motion paths [21,22]. These characteristics have clear benefits for applications in search and rescue; however, they also introduce a multitude of design complications. The high number of DOFs present in soft-bodied robots create greater challenges in proprioception [21] and accurate modelling, where small discrepancies can lead to significantly altered results [23,24]. These issues increase the demands on a control and sensor system. Combination robots aim to couple the desirable effects from both hard and soft robotics—exhibiting high degrees of freedom and compliance whilst adopting a hard exterior to prevent any external damage such as punctures from contact with sharp objects [25]. Within worm-inspired robotics, designs have often focused on singularly hard- or soft-bodied approaches. However, there are more examples of combination robotics inspired by the wider family of soft-bodied invertebrates, such as pentapods [25].

The use of bio-inspired robotics in search and rescue missions, such as rapid search response, accident investigation, and victim and evidence recovery, is a developing but wide-ranging field [26]. Robotics can be used to reduce risks (compared with the risk to life during human-led operations) [27], increase mission efficiency with quick deployment [26,28,29] and expand search times [30]. Models can be optimised for these applications with equipment such as a multi-beam imaging sonars [30] or chemical sensors [29], useful for identifying targets, especially in low visibility conditions. For victim recovery-focused missions, robots can be used to deliver supplies or communication equipment, especially during long-duration missions. The addition of ‘grabber arms’ could also aid robots to retrieve evidence [30]. Existing worm-inspired robots with intended search and rescue applications include a soft-bodied prototype developed by Stanford University [27]. The design consists of a tube of soft material folded inside itself that extends in one direction when the material at the front of the tube everts, and uses a control system to differentially inflate the body and change direction (basing the decisions on images sent from a camera). This demonstrates the success of pneumatically actuated soft-bodied robotics in these applications. A similarly long-bodied, eelworm inspired robot proposed by the University of Leeds [31] utilises serpentine locomotion. Notably, the design takes inspiration from snakeskin, with an outer shell that provides low-friction moving forward but high friction moving sideways or backwards.

Pneumatic artificial muscles (PAMs) are a class of pneumatic actuator devices specifically designed for the mimicry of biological muscles. First introduced in the 1950s, their use has spread in soft and mobile robotics, despite initially slow development to early designs [32,33]. PAMs resemble biological muscle in their linear contractions, decreasing load–contraction relations and inherently compliant behaviour [32,33]. The force-to-weight ratios achieved are significantly higher than motoric systems [32]. Due to their core element of a single membrane, PAMs can be lightweight, easily manufacturable, and replaceable compared with alternative actuation devices [32]. During pressurisation, the membrane’s volume increase is translated into a radial expansion and longitudinal linear contraction, creating an inward pulling force [33]. This volume increase is usually achieved by effective geometric design or high tensile-resistant materials. The variety of movements achieved with PAMs can be extended with ‘multi-chamber actuation’ [19], arranging the PAMs arranged in series, in parallel, in antagonistic configurations or directly built into a complex operational structure [34]. For example, a three-parallel PAM configuration would allow for three degrees of freedom motion. McKibben PAMs are one of the most frequently encountered PAMs in soft-robotics, owing to their simple design, easy assembly and low cost [32]. The McKibben design typically consists of an inflatable elastomeric tube, enclosed in a flexible braided sleeving [35]. The PAM’s volume increase during pressurisation is achieved via the expansion of the inner tube. When air is allowed to flow out, the sleeving acts as a spring to restore the tube to its original form [36]. Materials often used for McKibben PAMs include latex or silicone for the inner tube and Nylon fibre, silk, and steel mesh for the braided mesh [37]. The pleated PAM (PPAM) incorporates longitudinal indentations, or pleats, throughout the surface of the pneumatic actuator. When pressurised, the pleats unfold circumferentially, allowing for an increase in volume with no substantial stretch of the membrane. This reduces the risks involved with material strain; however, flexibility is still necessary to facilitate the unfolding [33]. The pleated design achieves greater efficiency, with larger contractions, in comparison to the McKibben PAM, as the increased surface area allows for greater contraction at the same gauge pressure [33,38]. Geometry-based actuators that contract and elongate (GRACEs) are a class of PAMs reported in a recent 2022 paper by De Pascali and co-workers [34]. The comparatively simple design consists of a single monolithic element: a thin membrane with longitudinal pleats and an engineered curvilinear shape. Similarly to the pleated PAM, the GRACE PAM contracts axially as the pleats unfold, without the substantial stretching of the membrane. The geometry inherently implements inflation to axial contraction, reducing the number of components needed, as operation does not rely on tensile-resistant components, end caps, or constraints present in previous PAM designs [34]. Under isotonic tests, GRACE PAMs have achieved load-to-weight ratios of up to thousands. Since the GRACE operating principle is based on the actuator’s geometry, they are manufacturable at a wide range of scales and with a wide range of materials, including low-cost three-dimensional (3D)-printed resins. Moreover, they can maximise the contraction, elongation, or antagonistic actuation by design. However, the rounded shape of the GRACE PAMs makes them larger than other PAMs of the same length, which for compact designs, may require scaling down and arranging several GRACEs in series [34].

In this paper, we hypothesise that peristaltic motion can be mimicked by a soft-bodied modular robot, consisting of four repeating units that are each individually pneumatically actuated by three artificial muscles. This hypothesis will be tested by designing, manufacturing and validating a design that is a **p**neumatically **a**ctuated perista**L**tic **a**dvancing **m**odular (PALAM) robot.

## 2. The Design Process

### 2.1. Overarching Concept

Three different types of PAMs were considered as possible actuation devices for the worm-inspired PALAM robot, to check the suitability of each, and allow for an optimal design choice to be made. The GRACE PAM is selected for implementation.

**Figure 1:**
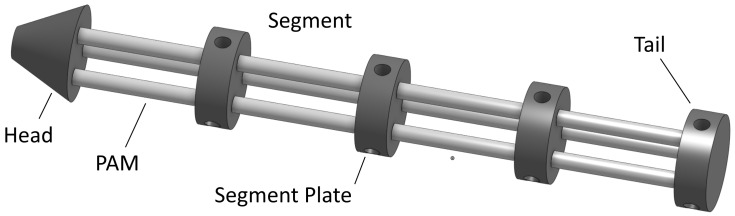
Symbolic diagram of the intended design.

The PALAM robot is based on a fully modular design consisting of individual segments, which can be assembled in linear sequence. The intended design for manufacture consists of four segments, as shown in Figure 1. Each segment consists of three pneumatic artificial muscles (PAMs), the air supply, and electronics necessary to route the pressurised air to the PAMs and a rigid connecting segment plate.

At the four segment plates, pressurised air will flow via the 12 solenoid valves into each of the PAM inlets. When all the PAMs in a segment are inflated, the segment will contract (and *vice versa*, deflation will cause elongation). In this way, the segment will work like sets of biological muscle, contracting and elongating to create peristaltic motion. Single PAMs in a segment can be individually contracted, altering the motion path and hence three PAMs were identified as the minimum needed to obtain a full range of up–down and left–right motion. A control system will be designed, with the aim of contracting any PAM in the robot on demand.

### 2.2. Dynamics and Kinematics

#### 2.2.1. Kinematics

**Figure 2:**
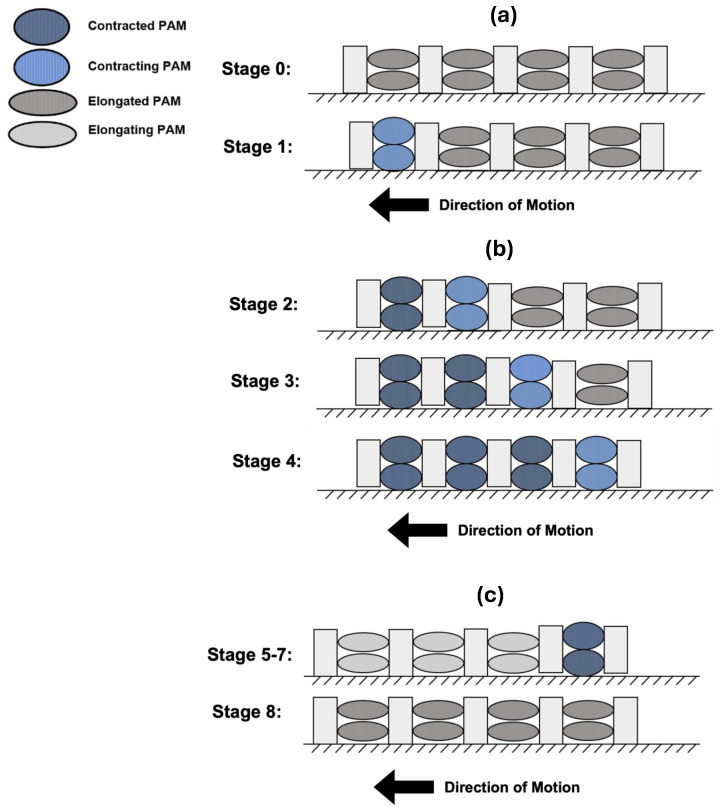
(**a**) The first stage of peristaltic motion (**b**) stages 2 to 4 of peristaltic motion and (**c**) the final Stage of peristaltic motion.

The peristaltic motion of the PALAM robot depends on the four segments contracting in sequence. Two approaches were considered for achieving peristaltic motion this way: the first relies on the PAMs to both contract and anchor themselves on the contact interface. The second involves the PAMs contracting while the segment plates perform the anchoring function. The former approach was selected for achieving peristaltic motion, as it allows for the worm to move in multiple directions (while the second is restricted to unidirectional movement). Moreover, the first model imitates the peristaltic motion of earthworms more closely, as the segments are able to perform both contraction and anchoring functions. The second motion model was only considered as a fallback due to the risk of potential limitations of the PAMs successfully providing sufficiently precise anchoring force (due to the soft-bodied nature of the PAMs). To achieve a complete cycle of linear peristaltic motion with the first motion model, eight stages are required. The robot at rest is considered to be at Stage 0, when all PAMs are deflated and each segment is at its rest length. The robot should make contact with the interface (terrain over which peristaltic motion is achieved) at the segment plates only. This is shown as Stage 0 in Figure 2. When all three PAMs in Segment 1 are inflated, contracting axially, and expanding in the vertical direction, the segment generates an inward pulling force, shown as Stage 1 in Figure 2a. Once Segment 1 is inflated, the two PAMs on the lower level make contact with the interface, so the robot is anchored with friction at this point of contact. Then, during Stage 2, as shown in Figure 2b, when Segment 2 is contracting, Segment 1 is anchored to the interface, and so Segments 3 to 4 are pulled along. Similarly, Segment 3 is contracted, pulling along Segment 4, and finally Segment 4 is contracted, resulting in the eventual maximum contraction of the robot. Crucially, the already contracted segments should not move towards the currently contracting segment, anchored by their contact with the interface, as shown in Figure 2b. Finally, Segments 1–3 are elongated in sequence (with the PAMs deflated and returning to their natural shape) while Segment 4 remains contracted. In this way, while Segment 4 is anchored to the contact interface, Segments 1–3 are pushed forwards, as illustrated in Figure 2c.

#### 2.2.2. Validation

**Figure 3:**
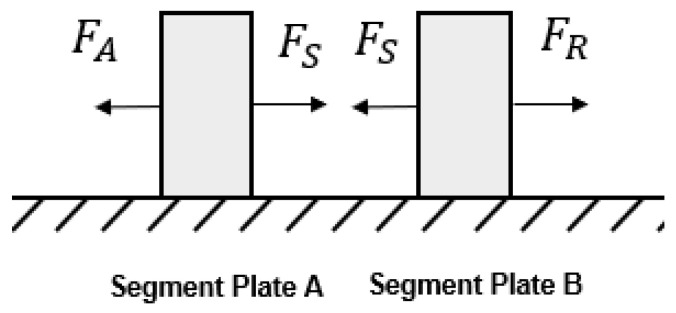
Static model during contracting stages.

A static model was initially used to determine the forces that must be overcome to achieve peristaltic motion. The generalised scheme of Figure 3 can be constructed for the contracting stages of peristaltic motion with the following key assumptions made:

1. While the PAMs in a segment are contracting, they act as a spring generating a uniform inward pulling force, denoted by $F_{S}$.
2. When the PAMs in a segment are contracted, the two PAMs low enough to touch the contact interface are anchored by friction, anchoring the segment at this point with force $F_{A}$.
3. The elongated segments generate resistive forces at the plates (as they are pushed or pulled along) that can be modelled as one resistive force to motion denoted by $F_{R}$.

In Figure 3, Segment Plate A, of mass $m_{SA}$, is the segment plate behind the most recently contracted segment. Segment Plate B, of mass $m_{SB}$, is the segment plate in front of the elongated segments. From this, it can be shown that, in order to achieve motion, the anchoring static friction force, $F_{A}$, must be verified as able to equal the inward pulling force of the segment, $F_{S}$ (noting that $F_{A}$ is a static friction force that will increase to prevent motion up to its maximum value). Additionally, the inward pulling force of the segment, $F_{S}$, must be verified as able to overcome than the static resistive force to motion of the elongated segments and plates, $F_{\left(R\right)}$. As $F_{\left(R\right)}$ is the lower inequality, the maximum potential value is important. This can be expressed in a single inequality:

(1) $$F_{A_{max}}\geq F_{s}>F_{R_{max}}$$

**Figure 4:**
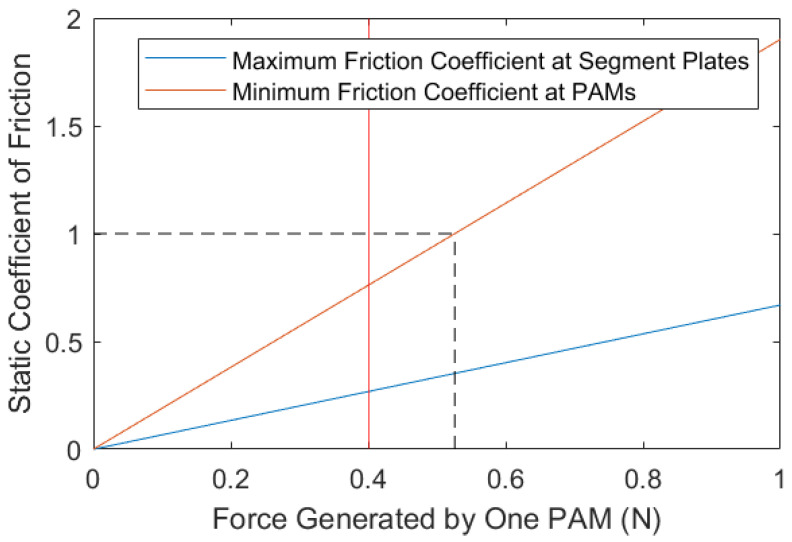
Friction coefficients for achieving peristaltic motion.

Clearly, to allow for a larger range of values for $F_{S}$, it is desirable to minimise $F_{A_{max}}$ and maximise $F_{R_{max}}$ (The calculations for $F_{A}$ and maximum $F_{R}$ are outlined in Appendix A.1). For motion to be achieved, the segment plate’s friction coefficient has to be less than the value at $F_{S}=F_{R_{max}}$ (referred to as the maximum coefficient at the plate) and the PAM’s friction coefficient has to be equal to or greater than the value at $F_{S}=F_{A_{max}}$ (referred to as the minimum coefficient at the PAMs). A MATLAB code was written to determine the coefficients necessary for varying $F_{S}$ values, while fulfilling Equation (1) and ensuring motion. Figure 4 shows this relationship between the force generated by a PAM and the resulting necessary coefficients.

From this, it can be established that the force of the PAM cannot exceed 0.5 N (assuming that friction coefficients greater than 1 cannot be reliably achieved over a range of terrains). If the force of the PAM is 0.4 N, for example, the maximum friction coefficient at the segment plates is 0.27 and the minimum friction coefficient at the PAMs is the friction coefficient 0.76. This could be achieved with segment plates manufactured from materials similar to polytetrafluoroethylene (PTFE) or polyethylene (as their friction coefficient ranges are less than 0.27), and with PAMs manufactured from materials close to rubber [39,40] (as their friction coefficients can be greater than 0.76). It can therefore be concluded that peristaltic motion is achievable with the described motion model.

#### 2.2.3. Actuator Requirements

The following requirements must be determined to theoretically prove the success of a PAM-actuated robot:

i FORCE—a PAM must generate sufficient force.
ii INFLATION—a PAM must inflate and contract reliably.
iii DEFLATION—a PAM must deflate and elongate reliably.
iv RIGIDITY—a deflated PAM must hold its shape.

Three different PAM types were considered throughout these design development stages—the McKibben PAM; the pleated PAM; and the GRACE PAM—and each of these are considered in the calculations below.

 **(i)**  **Force of PAM**

During inflation, the volume increase in each PAM segment is translated into an inward pulling force, $F_{PAM}$. During forwards peristaltic motion, when all three PAMs in a segment are contracted, the segment’s total inward pulling force is $F_{S}\left(x\right)$, as shown in Equation (2) ($F_{S}$ is established to be a function of contraction, *x*, in this section). For peristaltic motion to be achieved, $F_{S}\left(x\right)$ must overcome the maximum resistive force, $F_{R}$, acting on a segment at any one time, without overcoming the anchoring force, $F_{A}$, at any stationary contracted PAM (see Equation (1)). This section outlines the theoretical forces generated at controlled pressures to predict $F_{S}\left(x\right)$ values. These are not directly comparable with the forces used in the static model of Equation (1), but can be used to validate its success.

(2) $$F_{S}\left(x\right)=3F_{PAM}$$

A MATLAB code was developed to calculate the theoretical force generated for a range of PAM types and sizes (from the model outlined in this section), with varying contractions and employed to validate design decisions. It was concluded that forces necessary to fulfil Equation (1) can be easily achieved with a segment of three PAMs. While they have theoretical force capabilities (at ideal, constant pressures) that exceed 0.5 N, this may be significantly reduced experimentally, as the operating pressure will be inconsistent and limited by system losses. It is important to note, however, that during operation of the PAMs, the generated force may still need to be controlled to ensure this that limit is not overcome.

**Theoretical Model**

Multiple theoretical models for calculating the forces generated were considered, as outlined in Appendix A.2. The selected model was the Chou and Hannaford model (shown in Equation (3)), which allows force to be calculated without any consideration of the detailed geometry of a PAM, and demonstrates that increasing the contraction leads to a reduction in generated force. Quasi-static conditions and negligible energy losses (including energy required to deform or rearrange the membrane) are assumed [33,41].

(3) $$F=\rho \frac{dV}{dL}$$

where ρ is the pressure gauge in the PAM, dV is the change in the volume of air enclosed in the PAM, and dL is the contraction, that is, the change in the PAM’s axial length compared with its maximum value at deflation [32,33,41,42].

#### 2.2.4. Validation

**Figure 5:**
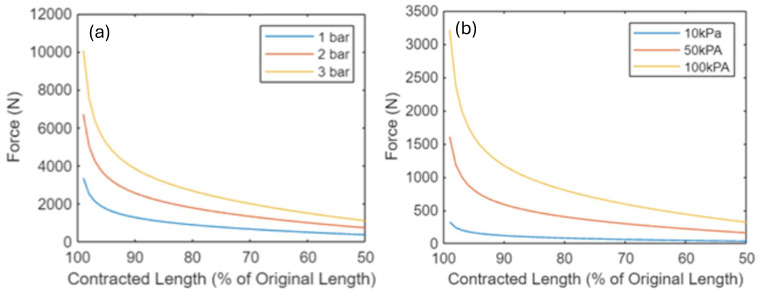
(**a**) Force–contraction Diagrams for a Pleated PAM of deflated length 0.11 m and diameter 0.0235 m operating at a range of pressures for comparison against theoretical data [41]; and (**b**) Force–contraction Diagrams for a Pleated PAM of deflated length 0.1 m and diameter 0.025 m operating at a range of pressures for comparison against experimental data [32]. Our MATLAB code developed using Chou and Hannaford model was outlined above following Equation (3).

The correct implementation of this model was verified through a comparison with established research. Figure 5a shows that the selected approach successfully produces theoretical values in line with established theory [41]. Figure 5b shows that the selected approach produces overestimated theoretical values compared to experimental data [32]. This is in line with the known limitations of the Chou and Hannaford model. The average error is an increase of around 50%, which can be taken into account when the model is employed to validate design decisions.

- At 50 kPa and 10% contraction, the experimental data show a force of around 400 N, while the MATLAB code calculates 584 N, which shows a 46% error.
- At 100 kPa and 10% contraction, the experimental data show a force of around 750 N, while the MATLAB code calculates 1170 N, which shows a 56% error.
- At 150 kPa and 10% contraction, the experimental data show a force of around 1200 N, while the MATLAB code calculates 1753 N, which shows a 46% error.

**Figure 6:**
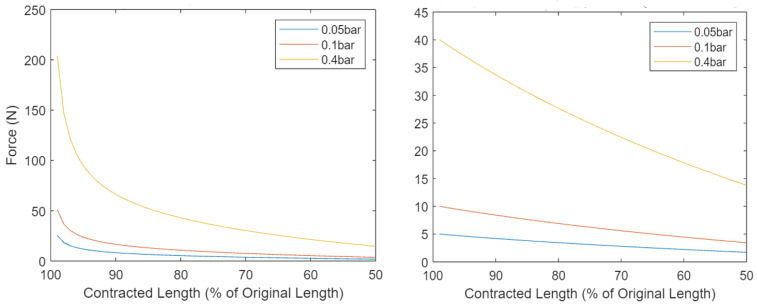
Contraction diagrams for a McKibben/Pleated PAMs of deflated length 0.04 m and an end diameter 0.016 m operating at a range of pressures.

Figure 6 shows the theoretical forces during contraction for the three different PAM designs considered (assuming constant idealised pressure). The results from this model show for PAMs with the same length and end diameter, GRACE PAMs will produce smaller theoretical forces than McKibben or Pleated PAMs. This is because the volume models have larger initial volumes for GRACE PAMs (reducing the change in volume and hence force). However, the GRACE PAM remains a suitable choice for use in the PALAM robot, due to their force requirements. For example, for the GRACE PAM design, with an operating pressure of 5 kPa, forces reach 5 N, falling to 4.2 N at 10% contraction (90% of original length).

 **(ii)**  **Inflation of PAMs and maximum operating pressure**

For the PAMs to operate, pressurised air is pumped into the membranes through a solenoid valve, creating the volume increase and axial contraction required. The operating pressure does not affect the contraction of a PAM; however, increasing the input pressures increases the output forces [34]. In order to select a suitable operating pressure for the PAM, the required force was calculated and the following existing research [32,34,42,43,44,45,46] into the McKibben, pleated, and GRACE PAM was utilised.

(a) *McKibben PAM*—The typical operating pressure range of a McKibben PAM is between 100 kPa and 500 kPa [32]. However, the McKibben PAM cannot generate both low and high forces [32]. The pressure the PAM can withstand is based on the material chosen for the inner tube [32]. If a softer rubber is chosen for example, the McKibben PAM can only operate at low pressures, as a pressure too high could cause the tube bulge through the mesh and cause it to burst [32]. Equally, if a tough rubber is used, the threshold pressure required to inflate the tube is high, often over 90 kPa [42], meaning lower forces cannot be produced. As for typical values of force produced, for a gauge pressure of 300 kPa around 220 N of pulling force can be exerted [46], although this value varies depending on the size of the PAM.
(b) *Pleated PAM (PPAM)*—The typical operating pressure of a PPAM ranges typically from around 10–100 kPA [32]. This is significantly lower than the McKibben PAM due to the absence of friction and the thin membrane [32]. The gauge pressure does have to be limited to these lower values because of the high radial expansion exhibited by the PPAM. However, even at a pressure of 100 kPa, forces of up to 1600 N can be produced [44].
(c) *GRACE PAM*—The 3D-printed flexible 80A resin GRACE PAM [34] works effectively at low pressures, producing a pulling force of 17 N force when pressurised at 22 kPa [34]. The maximum operating pressure is between 15 and 30 kPa for a resin GRACE PAM [34]. However, depending on the material choice, the force produced, and the operating pressure can vary. For example, a material such as FilaFlex 98A [47] has a maximum operating pressure of 220 kPa [34] and can produce greater forces.

 **(iii)**  **Deflation of PAM**

In order for a PAM to operate correctly, it is important that deflation occurs on demand, as this enables the axial elongation necessary for peristaltic motion. The McKibben design relies on the elasticity of the inner tube and the braided netting spring-like behaviour to restore the PAM to its original form [36], and so the yield strengths of the inner tube material should never be exceeded. The pleated and GRACE PAMs rely on their geometry to return to their original shape after inflation. From the inflation analysis above, the range of pressures at which existing PAMs designs operate correctly (including reliable deflation) can be used as a guide for ensuring the deflation of a manufactured PAM. Depressurisation would allow for an increased assurance of deflation [48].

 **(iv)**  **Rigidity of PAM**

During stage 5 of peristaltic motion, Segments 1–3 must provide sufficient tensioning force to remain elongated as this will allow the worm to advance forward. Experimentally, the rigidity of the PAMs can be evidenced by simulation and testing, and if insufficient, an additional spring can be added to the centre of the segments.

### 2.3. Material Design Theory

The successful operation of the PALAM robot relies on suitable selection and effective operation of materials. The PAM actuators in particular are reliant on material theory for successful operation, as their core element is a single membrane.

McKibben PAMs, consisting of an inflatable elastomeric tube enclosed in a braided sleeving rely on material properties such as elasticity [35]. The design faced several operational problems, many of which arose from material deformation and failure. For example, dry friction is inherent to the design [32], and the friction between the inner tube and the mesh material led to material deformation and early material failure at points of attachment [32] as well as leading to increased temperatures that altered the PAM’s behaviour and limited the precision of force and displacements. Additionally, the energy required to ensure the elastic deformation of the inner tube lowered the force generating potential of the PAM [32]. Ultimately, the material failures and limited life expectancies of McKibben PAMs led to future iterations of the PAM. The Pleated PAMs, in direct contrast, rely on materials of high tensile strength and stiffness for the membrane, relying on unfolding pleats instead of elastic deformation. This removed the risk of friction related hysteresis [33]. The pleated design also achieves greater efficiency in comparison to the McKibben PAM, as the increased surface area allows for greater contraction at the same gauge pressure [38].

The design for the GRACE PAMs allows for greater adaptability in terms of material choice, as the GRACE operating principle is based on the actuator’s geometry rather than material properties. For example, increasing the material stiffness does not significantly affect the contraction ratios, although it does lead to larger output forces and require higher input pressures for inflation [34].

### 2.4. Design Process: Summary

In summation, the five stages of motion must be completed for a complete cycle of peristaltic motion to be achieved by a worm-inspired robot with four actuated segments. Each of these stages rely on the three PAMs within each segment to simultaneously contract and hence produce an inward pulling force. The segment’s inwards force must fall within an acceptable range for successful peristaltic motion—that is, it must be sufficient to overcome the maximum resisting force on the segment, without exceeding the anchoring force of any other stationary contracted segments. In this way, it has been shown that the appropriate force range is determined by several factors, including the operating pressure, as well as the size, weight, and material of each part of the worm-inspired robot. A MATLAB Code has been developed to calculate this force range once these design parameters have been selected. From the assessment of the mathematical and material theory, the GRACE PAM presents the most suitable and effective actuator option. The operating principle allows for an adaptable manufacturing process, and a reduced restriction of material issues. Additionally, the range of operating pressures necessary are significantly reduced, relative to the McKibben and pleated PAM models. While the theoretical forces produced by the GRACE PAM according to the Chou and Hannaford model, this is a suitable choice for use in the PALAM robot, due to the force requirements.

## 3. Implementation and Analyses

**Figure 7:**
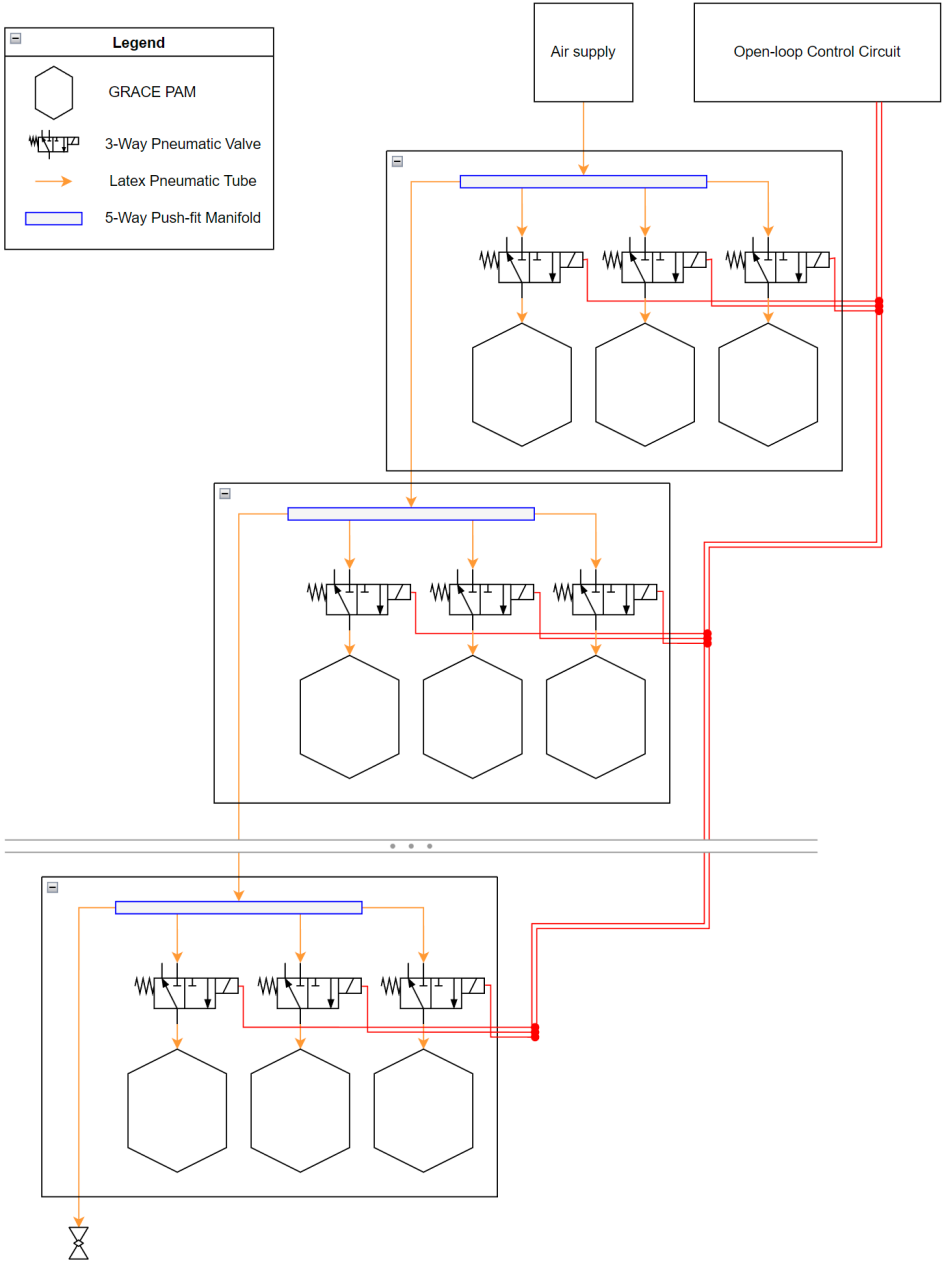
Full piping and instrumentation diagram of the PALAM Robot. It should be noted that there are four segments in total in the PALAM robot, with three PAMs connecting each segment.

Figure 7 illustrates our implementation of the design process considering the full design of the PALAM robot, followed by the steps taken to optimise and verify the designs before manufacture.

## 4. Actuator Design

A first prototype for a PAM design was developed (outlined in Appendix B.1). Critically, the lack of a well-defined geometry in this design led to difficulties in optimisation and ensuring successful operation. This was confirmed in testing, which revealed unreliable contractions. This validated the conclusion reached in the GRACE proposal paper [34], in that accurate geometry is crucial to an effective PAM design and the main determinant of the contraction ratio.

### 4.1. Geometrically Accurate Design

#### 4.1.1. The Original GRACE PAM Design (as Detailed in the GRACE Proposal Paper [34])

In the GRACE proposal paper [34], the PAMs are designed as a series of three curves per half-pleat, each defined by a quadratic function:

1. The Crest curve, for the top of the pleat;
2. The Edge curve;
3. The Valley curve, for the deepest point of the pleat.

**Figure 8:**
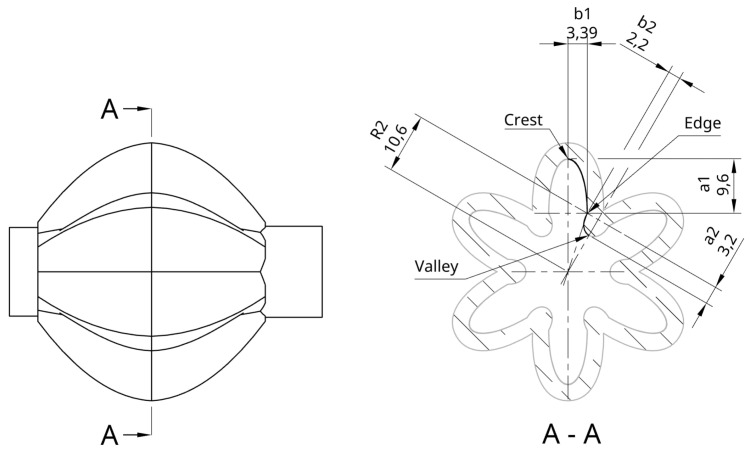
Central cross-section diagram of PAM, showing the 4 key parameters.

A surface connects these three curves while following two mathematically well-defined paths (which were sketched at the centre cross-section or CCS of the PAM). These paths are simple splines that connect the points of each curve at any given cross-section. Four independent parameters, as shown in Figure 8 and Table 1, are used to define the paths and curves. Note that these parameters are normalised and thus dimensionless; it is assumed that the same contraction ratio characteristics applies to any size of PAM as long as the ratio between the parameters remains constant.

The GRACE proposal paper [34] assesses the performance of the PAM with different configurations, using both numerical simulations provided in a MATLAB script and by manufacturing and testing a few select geometries. This was done for 4, 6, 8, and 10 pleat designs, with the paper concluding that there was no substantial difference in the performance between pleats, although the optimal parameter values were drastically different. Crucially, the profiles that perform better in contraction are generally those with narrow ellipses and deep pleats, which can expand further when the PAM is inflated.

#### 4.1.2. Implementation of the GRACE Design

The design for the PAMs in the PALAM robot utilised the data provided by the GRACE proposal paper [34]. The 6-pleat design was used, as this configuration appeared to be preferred, with more data and testing available compared to other pleat numbers. Additionally, as the PALAM uses three PAMs per module, a multiple of three for the pleat number allows for space between the PAMs and the interface in the full assembly while at rest. This is achieved with aligning of the PAMs to ensure that the “valley” of the pleat is facing the flat surface of the segment plate.

**Figure 9:**
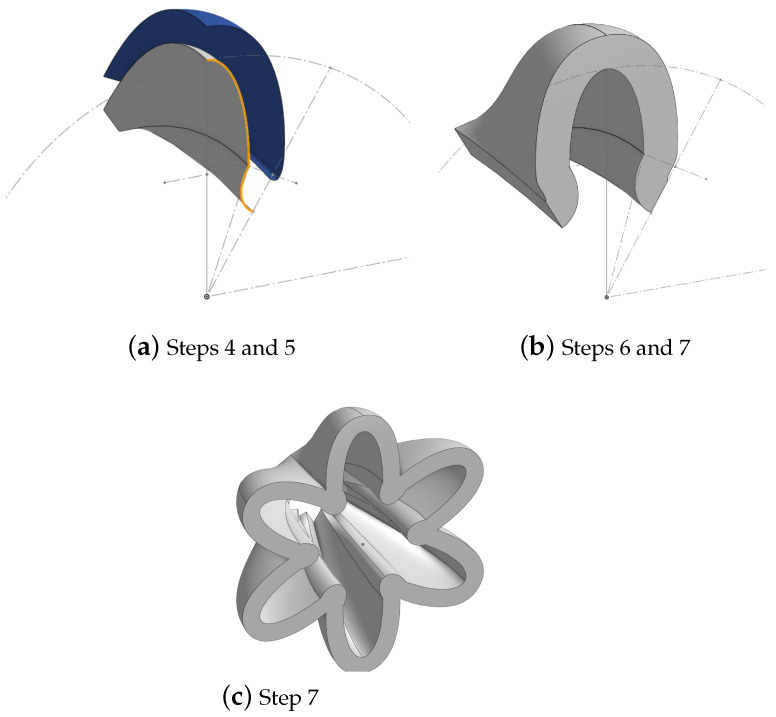
PAM CAD modelling process.

**Figure A2:**
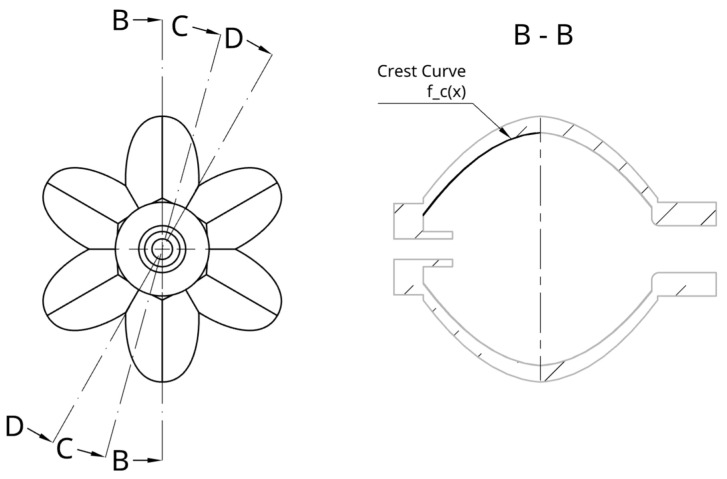

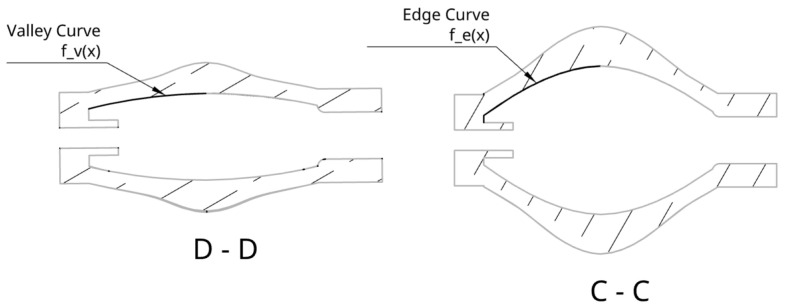
Diagram showing the three parabolic curves that the loft surfaces were generated between. The coefficients of the quadratic functions depend on the outer radius of the PAM connector and the CCS parameters, and were generated by a MATLAB code inspired by the GRACE proposal paper. Each function is defined so that each curve starts at the PAM connector and ends at their respective point on the CCS.

The GRACE proposal paper [34] provided a MATLAB script, detailing the generation of the parabolic curves. To generate these coefficients for the PALAM robot’s PAMs, a new MATLAB script was written, inspired by the mathematics in the provided code (see Figure A2). A PAM of 40 mm length was selected as the optimal balance between manufacturability and reduced size. All the given parameters from the GRACE proposal paper [34] in Table 1 and the derived functions were multiplied by this length, the PAMs modelled in CAD with the following steps:

1. Variables were created to store the CAD parameters (including the geometry parameters, number of pleats and curve function coefficients) allowing for scalability of the design if needed.
2. From these dimension constraints, the main CCS sketch was created. A half-pleat was modelled using splines between the curve points.
3. Using the parametric curve custom feature, the functions were plotted on the main plane. Then, using the angles of each point with respect to the vertical crest point, the curves were rotated to match their position on the PAM. The measurement feature was used to ensure that the rotation was automatically performed following a parameter change.
4. With the curves in position and aligned with the CCS points, the surfaces were created with the loft tool. The tool was configured to generate surfaces between the curves while following the sketch paths (this is the grey surfaces in Figure 9a).
5. To generate the thickness of the PAM, a new sketch was created with identical paths but with the points radially offset by the thickness variable. The transform feature was used to duplicate each curve and also offset them radially. A new surface was created again (the blue surface in Figure 9a).
6. Next, the fill feature was used to seal all the edges of the two surfaces, which enclosed them into an actual part rather than just a surface (i.e., a solid shape with a volume).
7. The PAM was finalised by mirroring the half-pleat to create a full pleat (see Figure 9b), then using a circular pattern to multiply this by the number of pleats needed (Figure 9c) and creating an accurately enclosed structure.
8. Finally, the interfaces were added using extrudes on either side.

The PAM hook connector’s inner diameter facilitates the melting of inner mould and matches the 6 mm hooks. The valve interface’s 3 mm inner diameter matches the valves outlet fitting. Both connectors feature a 16 mm outer diameter, making them significantly thicker than the PAM itself. This was to facilitate manufacturing and to the avoid premature failure of the actuators, as the GRACE paper FEA showed a stress concentration in the inward pleats near the two extremities.

## 5. Segment Design

**Figure 10:**
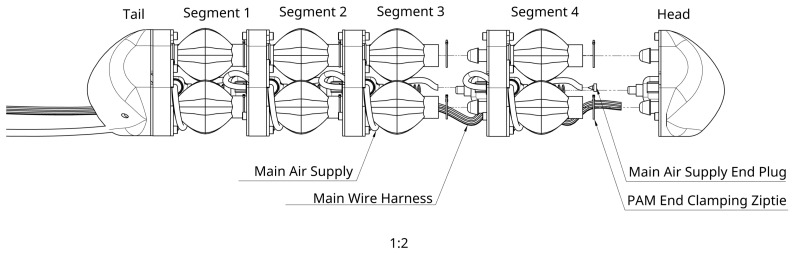
Side view of full PALAM robot assembly, showing one segment in exploded view. This illustrates the modularity of the robot.

The PALAM robot has a fully modular design consisting of individual segments, Figure 10. One segment consists of 3 PAMs, the pneumatic tubing necessary to route the pressurised air, valves to control the air flow into the PAMs and a rigid segment plate to hold the assembly in place. The pneumatic and electronic systems were designed for expandability, with an air supply line running across the entire robot.

### 5.1. Criteria and Component Selection

**Figure 11:**
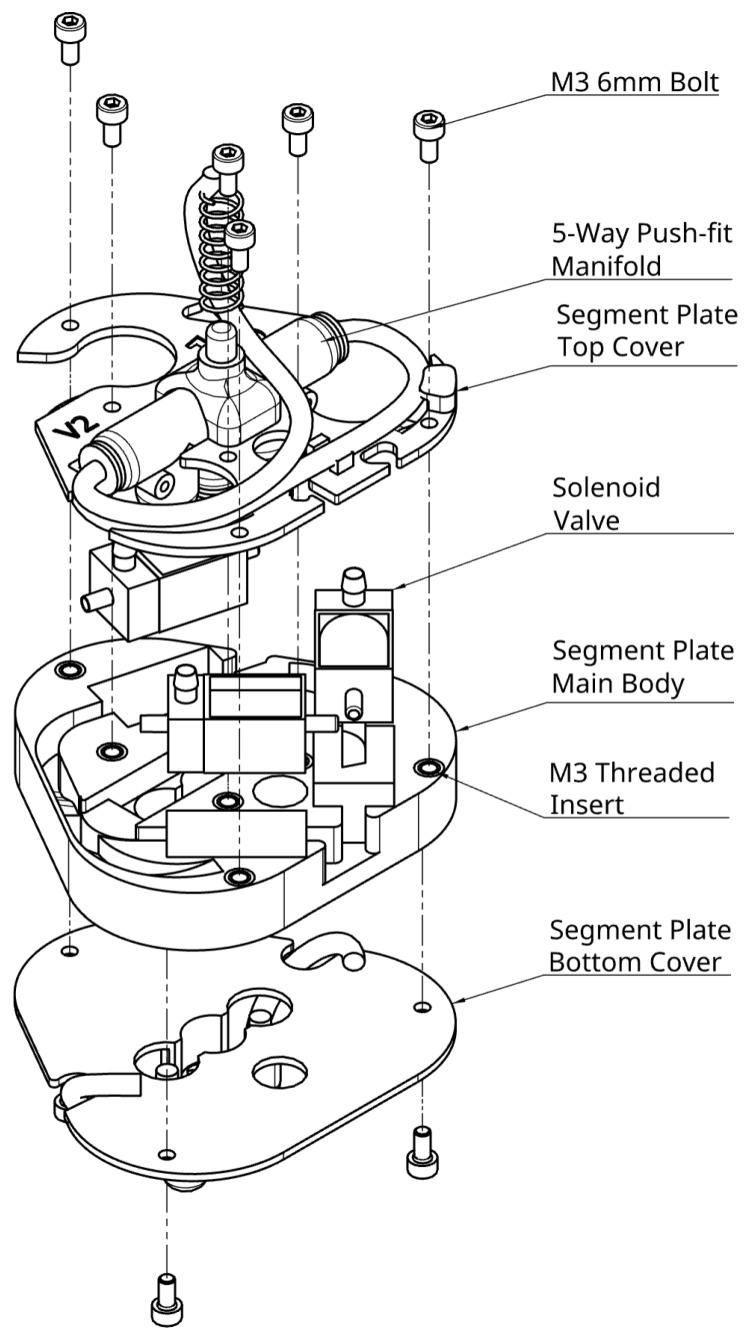
Exploded view of segment plate assembly.

Before the segment plate was designed, the components of the pneumatic circuit were selected (see Figure 11). Requirements for a successful pneumatic circuit design include:

1. Low resistance in the main air path, to ensure all PAMs can inflate at approximately equal rates regardless of their location, and maintain constant working pressure.
2. Reduced component size, to allow the perimeter of the segment plates to be smaller than that of the contracted (radially expanded) PAMs and thus for an increasing overall diameter of the PALAM robot as the PAMs contract.
3. Reduced thickness of the segment plates, to maximise the length occupied by the PAMs and maximise the worms speed.
4. Valves that withstand the working pressure.

#### 5.1.1. Tubing

The tubing was carefully selected to balance the need for both low resistance and compactness. A large enough inner diameter (ID) was needed, but the wall needed to be thick enough to withstand any bending. Multiple tube sizes were considered, from 3 mm to 8 mm outer diameter (OD) tubing. Due to the valves catering for large diameter tubing being too large, the focus was put on 3 mm and 4 mm OD tubing. For the outer diameter, 4 mm was selected, as a standard compact size that allowed for a reasonable flow rate at the working pressures. The inner diameter was less important for fitting compatibility, but crucial for flowrate. Initially, 3 mm ID was chosen to maximise the latter. However, due to the tight bends necessary to maintain a compact segment plate design, the thickness of a 3 × 4 mm tubing proved to be inadequate to prevent kinks, and 2 mm ID tubing was selected.

The material was also important for the tubing, both for flexibility and impermeability on push-fit fittings. It was found that latex rubber was more effective than the more standard silicone rubber at preventing kinks and sealing on push-fits.

#### 5.1.2. Five-Way Manifold

To split the air supply line amongst all PAMs, we used a manifold with five ports: one inlet, three for the PAMS, and one to supply the next segment. The options for this part were limited due to the size, tube diameter, and port number requirements. A low-cost, standard 5-way push-fit was thus selected.

#### 5.1.3. Solenoid Valves

Reduced size was the main constraint for selecting solenoid valves. Two types were considered:

1. Two-port, open/closed valves: These were available in smaller sizes, and were easy to mount and operate. Two would be needed per PAM to control inflation and deflation, adding the ability to precisely control the contraction size at the cost of space.
2. Three-port, two-way valves: These were equipped with an exhaust port for the PAM to deflate when the valve was closed. Only one would thus be needed per PAM, though they came in slightly larger sizes.

Both valves had a similar maximum pressure-to-size ratios. This meant that the 2-port valves, while smaller, could withstand less pressure. However, it was ultimately found that even the smallest available valves would easily withstand the working pressures utilised (see Section 2.2.3). The final selection of the valves was made based on complexity and size; three-port valves allowed for a significant space saving.

### 5.2. Segment Plate Design

Compact positioning of the components was important for the design of the segment plate, while maintaining the ease of assembly at small sizes.

#### 5.2.1. Component Placement

**Figure A3:**
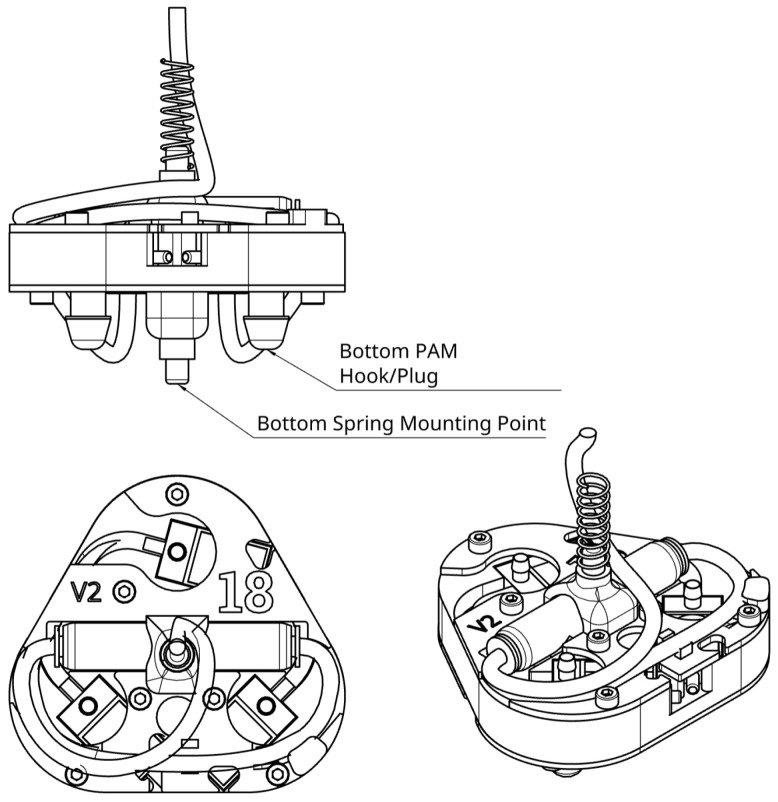
Segment plate assembly, showing PAM plugs, and spring mounts.

Three-dimensional CAD models of the main components were created and placed together in an empty assembly. The PAMs were mated to the valves to visualise the spacing. The manifold was significantly larger than other components, so needed to be embedded into the plate and kept central, to allow the top to “stick out” adjacent to the PAM interfaces at the non-contracting, “neutral” mounting point of the PAMs. The valves were then arranged as shown in the top view of Figure A3. This placement was validated by placing the physical components in the same way and connecting the tubing, to ensure feasibility and limited kinking.

#### 5.2.2. CAD Model

**Figure 12:**
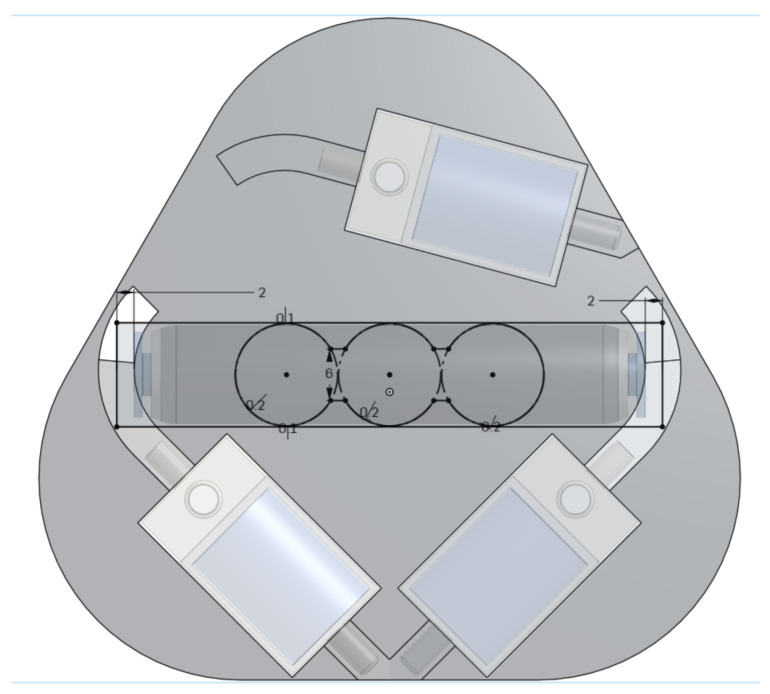
Extrusion being sketched in context.

With the components organised, in-context editing was used to create new parts from the assembly. First, the main shape was sketched and extruded into the main body part, to match the height of the valves, Figure 12. From this, the components were used as dimension references to sketch extrusion points from the shape to create holes where the components could sit.

Then, two additional parts were made: The bottom cover was modelled with 6 mm PAM barbed plugs (see Figure A3, allowing for the top hole of the PAM to be plugged and secured. The top cover secured the motors and part of the tubing in place. Having extra parts allowed the main body to feature various channels to route the tubing, securing and concealing it. The covers both feature sprint mounting points to allow for the adjustment of the tension or compression resistance of the PAMs, to account for any performance characteristics of the materials selected for their manufacture.

### 5.3. Fasteners and Mounting

Fastener selection was crucial to the assembly of the segment. Mounting holes were added after modelling the segment plate parts. These consisted of 3 mm holes on both covers and matching 4 mm holes in the body to allow for M3 threaded inserts to be fitted. M3 was the chosen size as it was readily available, small enough and easy to handle during assembly. The PAMs would be secured to the valves and barbed plugs using 2 mm zip-ties. These were selected due to their very low cost, ease of use, the and adjustability of the fastening force. However, in future prototypes, it could be advisable to use tubing clamps instead. Finally, the tubes were simply fitted to the valves by pushing them in, as this created an adequate seal for the low working pressures, and the main body channels would keep them secure. They would then be pushed into the push-fits.

## 6. Component Analysis

To validate the design, relevant CAD models were imported into SimScale FEA software, and a finite element analysis (FEA) was conducted, considering the worst-case scenarios of forces and pressures experienced by components. Components were modelled in the four inflated/deflated scenarios, each of which was found to withstand each other.

The component masses and material properties used are detailed in Table A1 and Table A2 in Appendix B.3. Isotropic material behaviour is assumed throughout. This is applicable to PAMs fabricated from silicone rubber, an elastomer that exhibits isotropic behaviour [49], although manufacturing imperfections may introduce some anisotropic behaviour. On the other hand, segment plates fabricated from PLA would have anisotropic behaviour [49], although the anisotropy is mild and for the inelastic response, it displays a ductile and orthotropic behaviour [12]. Throughout the scenarios, the materials never reach the stresses to display inelastic responses, so this is neglected [50]. As for the elastic response, the level of anisotropy is so small that it can safely be approximated as an isotropic material [11].

### 6.1. PAMs and Cable Ties

**Figure 13:**
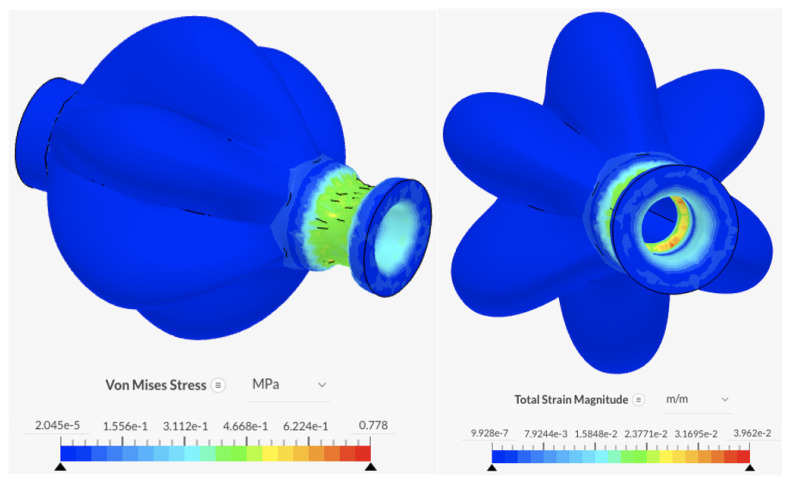
Von Mises stress and total strain magnitude in Scenario 1.

Cable ties were used to secure the PAMs to the solenoid valve and ensure air tightness, which generates a compressive force on both the PAM and valve. This was modelled as a pressure on the contact area between the PAM and the cable tie, with a force of approximately 100N. Fixed support boundary conditions were placed on the inner wall of the entry holes. Figure 13 shows the results of the analysis. The forces do not exceed the elastic limit of silicone rubber.

### 6.2. PAMs

**Figure 14:**
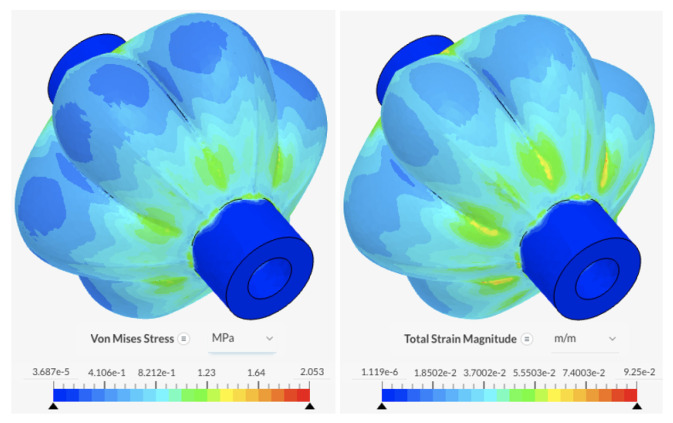
Von Mises stress and total strain magnitude in Scenario 2.

The state of maximum inflation, defined here as the state of inflation at 40 kPa, is simulated with fixed boundary conditions at the inner wall of the entry holes. The results in Figure 14 show the main body portions of the PAMs experiencing the highest levels of stress and strain, with values reaching 2.05 MPa and $9.25\times 10^{-2}$ m/m, respectively. The forces do not exceed the elastic limit of silicone rubber.

### 6.3. PAMs, Solenoid Valves, and Segment Plates

**Figure 15:**
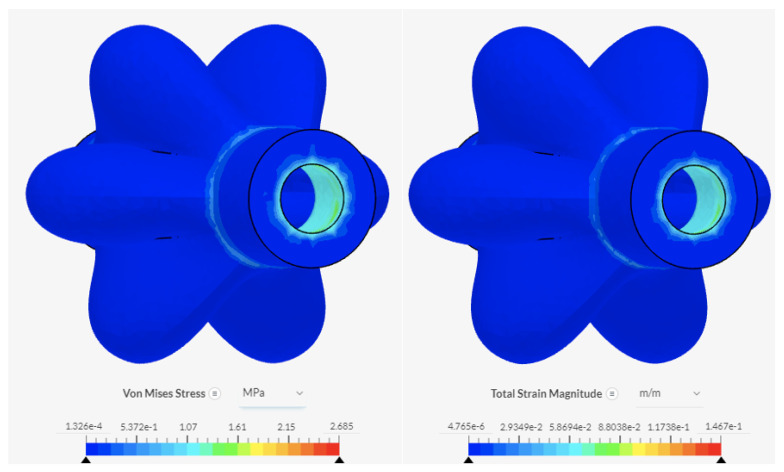
Von Mises stress and total strain magnitude in Scenario 3.

**Figure 16:**
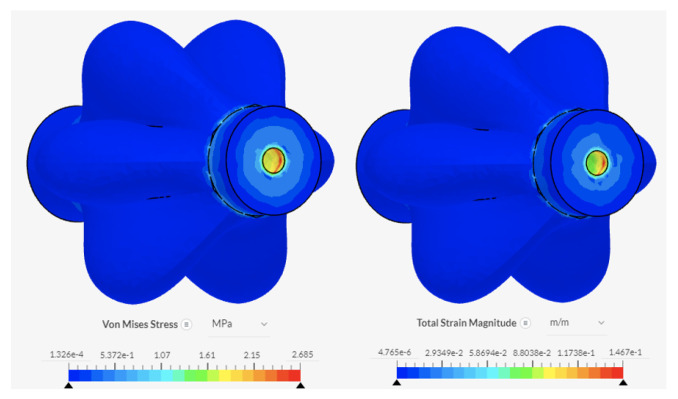
Von Mises Stress and total strain magnitude in Scenario 3 (facing the valve).

Forces experienced by the full assembly include the tension and compressive forces during elongation and contraction of the PAMs (experienced at the areas of the valves secured to the PAMs and the plates), and the friction forces between the segment plates and the interface. Additionally, a spring mounted in the centre of the segment plate would provide a resistive force when the PAMs are inflating. The worst-case scenario is identified to be without the additional spring, and with the largest frictional resistive force expected, as outlined in Equation (A3) and calculated to be 2.12 N. The force generated by the PAMs selected for modelling was 35 N (value at 40 kPa and 10% contraction from Figure 6). The membrane of the PAMs is identified as the limiting factor, with the lowest elastic limit of all components. Fixed-support boundary conditions were applied to the areas where the PAMs were held in place. Figure 15 and Figure 16 show the results. The smaller hole experiences the highest values of stress and strain at 2.69 MPa and $1.47\times 10^{-1}$ m/m, respectively, as the forces are spread over a smaller area. The forces do not exceed the elastic limit of silicone rubber.

### 6.4. Segment Plates

**Figure A4:**
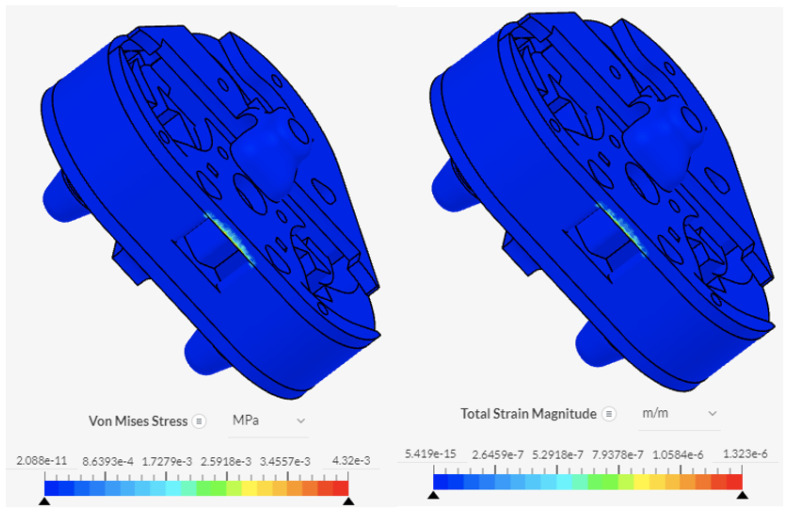
Von Mises stress and total strain magnitude in Scenario 4.

When the segment plates carry the load of the PALAM robot, the normal forces can be calculated (using Equation (A1) and mass values from Table A2). Fixed-support boundary conditions were placed on the bottom face and a normal force boundary condition on the face in contact with the ground. Figure A4 shows the results, with force and strain magnitudes at $4.32\times 10^{-3}$ MPa and $1.32\times 10^{-6}$ m/m, respectively, from which it can be concluded that the segment plates will withstand the weight of the worm.

## 7. Dynamic Modelling

Following the confirmed design, equations of motion were established for all stages of motion in the first motion model for peristaltic motion. The force generating part of the segments (the 3 PAMs) were modelled differently for each of their four modes: contracting, contracted, elongating, and elongated. Step functions are used to ensure that the segment is acting in the correct mode at the correct stage.

1. While contracting, the three PAMs in a segment can be modelled as a spring with stiffness $k_{C}$ generating a uniform inward pulling force. This stiffness $k_{C}$ can be determined from the theoretical force calculations, $k=k_{C}=\frac{F_{seg}}{x_{s1}}=\frac{3(F_{pam})}{x_{1}-x_{2}}$.
2. While contracted, the segments are anchored through friction between the PAMs and the interface. The segment can be modelled as a dampener, with resisting motion force $Fr_{s}$.
3. While elongating, the segment can be modelled as a spring with stiffness $k_{E}$ generating a uniform outwards pushing force.
4. While elongated, the segment can be modelled as a spring with stiffness $k_{e}$ generating a uniform outwards pushing force.

**Figure 17:**
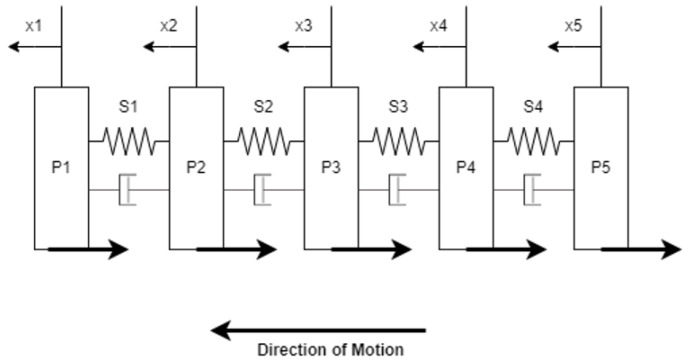
Free body diagram of PALAM robot, consisting of 4 segments and 5 segment plates.

Hence, in the free body diagram, shown in Figure 17, the segments were modelled with a spring of varying stiffness and a dampener. The friction force at the segment plate was modelled as a generalised force $Fr_{P}$ at the contact interface of each plate.

A key assumption made is that PAMs do not make contact with the ground until they reach their maximum contraction. Therefore, only PAMs in contracted segments are anchored with the ground. If additional support springs are added to the assembly, these can be accounted for by changing the stiffness of the springs modelling the segments. For example, when $k=k_{c},\;k_{c}=\frac{F_{seg}}{x_{s1}}=\frac{3(F_{pam})}{x_{1}-x_{2}}+\frac{F_{addspring}}{x_{1}-x_{2}}$.

Using Lagrange’s equation, $\frac{d}{dt}\left(\frac{\partial T}{\partial {\dot{q}}_{i}}\right)-\frac{\partial T}{\partial {\dot{q}}_{i}}+\frac{\partial V}{\partial {\dot{q}}_{i}}=Q_{i}$ the following equations of motion for each of the five segment plate masses have been produced. The calculations for the equations of motion are given in Appendix B.4.

E.O.M. for mass 1: $m_{p}\ddot{x_{1}}+k_{1}x_{1}-k_{1}x_{2}=-SA_{1}\left[Fr_{s}\right]-SB_{1}\left[Fr_{P1}\right]$

E.O.M. for mass 2: $m_{p}\ddot{x_{2}}-k_{1}x_{1}+\left(k_{1}+k_{2}\right)x_{2}-k_{2}x_{3}=-SA_{1}\left[Fr_{s}\right]-SA_{2}\left[Fr_{s}\right]-SB_{2}\left[Fr_{P2}\right]$

E.O.M. for mass 3: $m_{p}\ddot{x_{3}}-k_{2}x_{2}+\left(k_{2}+k_{3}\right)x_{3}-k_{3}x_{4}=-SA_{2}\left[Fr_{s}\right]-SA_{3}\left[Fr_{s}\right]-SB_{3}\left[Fr_{P3}\right]$

E.O.M. for mass 4: $m_{p}\ddot{x_{4}}-k_{3}x_{3}+\left(k_{3}+k_{4}\right)x_{4}-k_{4}x_{5}=-SA_{3}\left[Fr_{s}\right]-SA_{4}\left[Fr_{s}\right]-SB_{4}\left[Fr_{P4}\right]$

E.O.M. for mass 5: $m_{p}\ddot{x_{5}}+k_{4}x_{4}-k_{4}x_{5}=-SA_{4}\left[Fr_{s}\right]-SB_{4}\left[Fr_{P5}\right]$

The step functions used in the equations of motion are as follows:

$$\begin{array}{cc} & SA_{1}\left(t\right)=\left\{\begin{array}{cc} 0 & 0\leq t<2\\ 1 & 2\leq t<5\\ 0 & 5\leq t\leq 8 \end{array}\right.\;SA_{2}\left(t\right)=\left\{\begin{array}{cc} 0 & 0\leq t<3\\ 1 & 3\leq t<6\\ 0 & 6\leq t\leq 8 \end{array}\right.\;SA_{3}\left(t\right)=\left\{\begin{array}{cc} 0 & 0\leq t<4\\ 1 & 4\leq t<7\\ 0 & 7\leq t\leq 8 \end{array}\right.\\ & SA_{4}\left(t\right)=\left\{\begin{array}{cc} 0 & 0\leq t<5\\ 1 & 5\leq t<8\\ 0 & t=8 \end{array}\right.\;SB_{1}\left(t\right)=\left\{\begin{array}{cc} 1 & 0\leq t<2\\ 0 & 2\leq t<5\\ 1 & 5\leq t\leq 8 \end{array}\right.\;SB_{2}\left(t\right)=\left\{\begin{array}{cc} 1 & 0\leq t<3\\ 0 & 3\leq t<5\\ 1 & 5\leq t\leq 8 \end{array}\right.\\ & SB_{3}\left(t\right)=\left\{\begin{array}{cc} 1 & 0\leq t<4\\ 0 & 4\leq t<6\\ 1 & 6\leq t\leq 8 \end{array}\right.\;SB_{4}\left(t\right)=\left\{\begin{array}{cc} 1 & 0\leq t<5\\ 0 & 5\leq t<7\\ 1 & 7\leq t\leq 8 \end{array}\right.\;SB_{5}\left(t\right)=\left\{\begin{array}{cc} 1 & 0\leq t<5\\ 0 & 5\leq t<8\\ 1 & t=8 \end{array}\right. \end{array}$$

The maximum resistive force on a segment occurs during Stage 2 on the contracting Segment 2.

E.O.M. for mass 1: $m_{p}\ddot{x_{1}}=-Fr_{s}$

E.O.M. for mass 2: $m_{p}\ddot{x_{2}}+k_{c}x_{2}-k_{c}x_{3}=-Fr_{s}-Fr_{P2}$

E.O.M. for mass 3: $m_{p}\ddot{x_{3}}-k_{c}x_{2}+\left(k_{c}+k_{e}\right)x_{3}-k_{e}x_{4}=-Fr_{P3}$

E.O.M. for mass 4: $m_{p}\ddot{x_{4}}-k_{e}x_{3}+\left(2k_{e}\right)x_{4}-k_{e}x_{5}=-Fr_{P4}$

E.O.M. for mass 5: $m_{p}\ddot{x_{5}}-k_{e}x_{4}+k_{e}x_{5}=-Fr_{P5}$

A MATLAB 2019a script was used to solve the system of equations of motion numerically. This described the motion of each mass, and the velocity of the system. Depending on the materials selected and the design specifications, different values for $k_{c}$, $k_{e}$, $k_{E}$, $Fr_{s}$ and $Fr_{p}$ could be adopted. $k_{c}$, $Fr_{s}$ and $Fr_{p}$ are theoretically established (see MATLAB Code), and $k_{e}$ and $k_{E}$ can be established experimentally. The inflation time was assumed to be 1 s in this section; however, the step functions can be updated when the actual inflation time of the PAMs is measured after fabrication.

## 8. Electronics and Control System

The control system is designed to individually command the contraction and elongation of each PAM. This section details the implementation of electronics designed to match the actuation system outlined in the Piping and Instrumentation Diagram in Figure 7, and presents a more advanced PID controller design possibility.

### 8.1. Valve Switching Circuit

The solenoid valve consists of a coil which creates an electro-magnet, and a spring-loaded metal plunger. This coil operates at 6 V and draws about 100 mA of current when energised. It was found that, however, during testing, the valve is easily actuated at voltages as low as 4 V. Due to the operation voltage of most MCUs (including the Arduino Uno and Due that were used) being 5 V, this was the chosen operating voltage for the valve. The 100 mA current is higher than most integrated circuits (IC) can handle. Therefore, it was necessary to add a metal–oxide–semiconductor field-effect transistor (MOSFET) switching circuit. This consists of a 2N7000 signal (i.e., low-voltage) MOSFET [51] with a pull-down resistor at its gate terminal. When the gate is pulled to a logic HIGH (i.e., 5 V is applied to it), the MOSFET starts conducting current, activating the valve.

### 8.2. Open-Loop Control System

**Figure 18:**
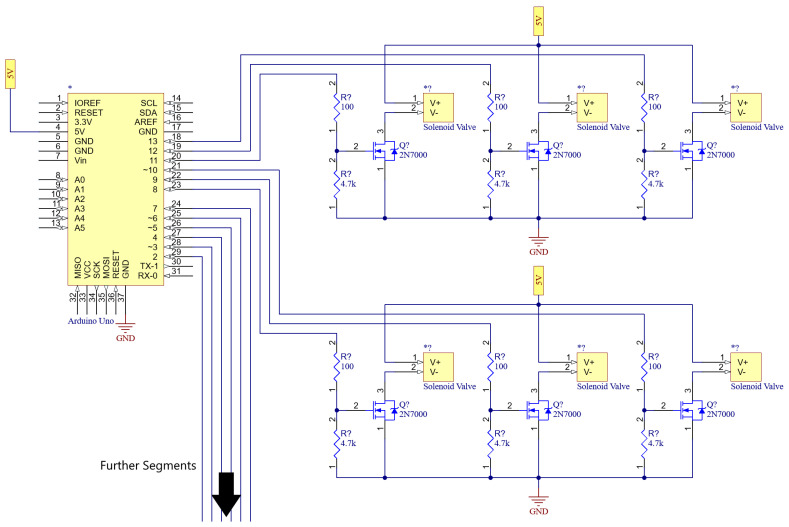
Electrical schematic diagram of the assembled open-loop controller system for the PALAM Robot.

As shown in Figure 18, a simple, open-loop controlled circuit was designed for implementation, to enable the testing of the final PALAM robot sufficient to confirm our hypothesis. Being open-loop, the circuit uses fixed timings to actuate the valves in sequence (the main motion algorithm). A micro-controller unit (MCU), in this case, an Arduino Uno or Due, can be programmed to activate the transistors with this sequence. By tuning the timing in the compiled code, the worm motion can be altered. The circuit can be implemented on a breadboard, with the valve negative wires (V− terminal of the solenoid valves in Figure 18) running from the breadboard to the individual wires on the worm. The circuitry on the right side of the figure represents the worth of individual segments, while the MCU and its outputs are shown on the left side.

### 8.3. Adding Feedback and Improving Modularity

A feedback-based, close-loop system using a PID loop mechanism was designed to improve the control of the PALAM robot, and hence its speed and efficiency. This is because the PAM contraction times are expected to vary wildly depending on load, position on the robot (due to tube resistance with longer robots), and other conditions. With an open-loop system, the timing must be tuned to allow the PAMs to always maximally contract, which introduces delays under low-load conditions. Furthermore, a closed-loop system allows for partial PAM contraction, something not possible within the timing tolerances of open-loop control. Partial PAM contraction is essential to achieving precise worm curvature, in order to ultimately achieve the three degrees of motion between segments. Such a system would allow for the precise directional control of a crawling worm and aiming any accessories attached (such as if panning a camera).

#### 8.3.1. Sensors

The main feedback needing to be implemented would be linear displacement, to track the PAM’s contraction. Using displacement feedback on each PAM would allow the controller to know the exact length and curvature of the worm, as well as the length of each individual segment. Displacement sensing can be performed using a variety of sensors, detailed in the literature review. Hall-effect [25], laser, and linear potentiometers are all effective solutions, given the small magnitude of PAM contraction. This design is based around linear potentiometers such as the Bourns linear motion potentiometer [52], as they are the easiest to implement both electronically thanks to its simple, linear analog voltage output (which easier to multiplex), and mechanically, thanks the fixed mounting points.

Pressure sensors are also useful to providing feedback, as the air pressure is directly correlated to the PAM inflation time and actuation at the contraction force. Having a pressure sensor at the main manifold allows the controller to adapt the PID algorithm accordingly. A small, printed circuit board (PCB) mounted pressure transducer such as the MPX4250 [53] can be used. Furthermore, by knowing the PAM force-contraction characteristics, one can measure the actuation time to accurately measure the forces exerted on the PAMs. This can provide insightful data during testing.

#### 8.3.2. Improving Modularity

A major disadvantage of the simple design is the clutter caused by the wires needed to control each PAM. With four segments, there would be 12 PAMs in the system, meaning up to 13 wires (12 wires plus the common 5 V, V+ wire) must enter the robot. Adding more segments and sensors dramatically increases the wire count. For instance, having 3 PAMs, 3 position sensors, and 8 segments would require a total of 49 wires.

**Figure A5:**
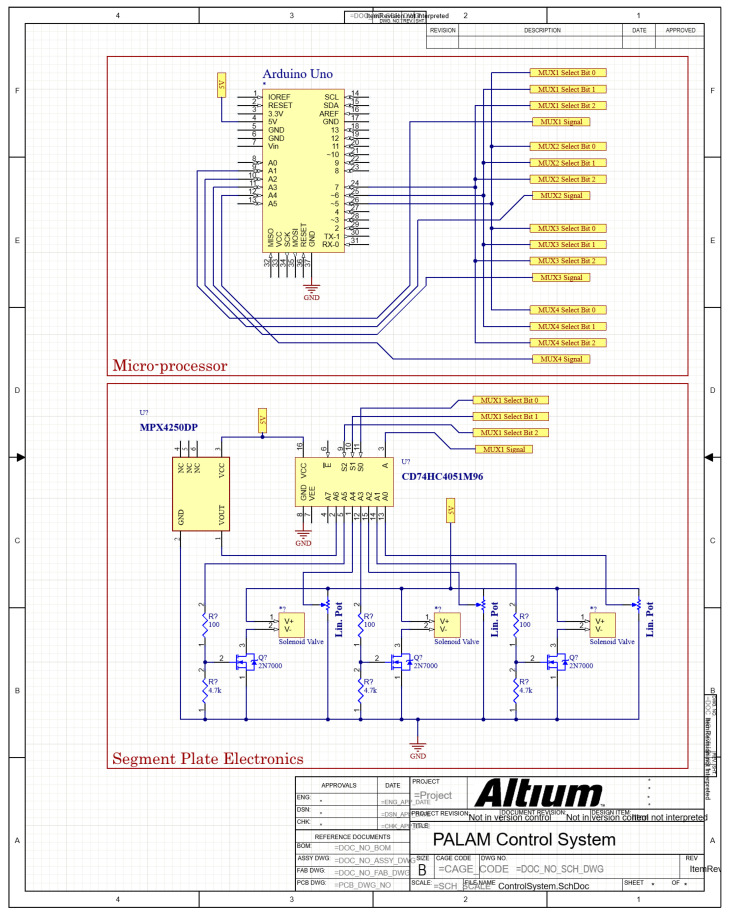
Electrical schematic diagram of a more advanced, closed-loop system for a 3-segment worm robot. The top half of this schematic shows the controller circuitry (outside of the segments). The bottom half represents the circuitry in the segments plates. The key components of the schematic are: The Arduino Uno, a micro-processor, a 3-to-8 multiplexer/demultiplexer (**Texas Instruments CD74HC4051** [54]) PCB-mounted, 20–250 kPa pressure transducer (**MXP4250** [53]) 5 kΩ sliding linear motion potentiometers (**Bourns 3046** [52]).

Using a series of multiplexers-decoder ICs (hereinafter referred to as multiplexers), the wire count can be decreased significantly. Multiplexers allow the transfer of data with multiple devices across a single wire using input selection wires, common to all segments, to select the input. Thus, by rapidly cycling through the inputs, the MCU can effectively write data or read values from many devices in a short time-span. Input selection is completed using simple binary signals on the selection wire; thus, the number of devices per multiplexer (so, per segment) $n_{D}$ is given by the number of selection wires so that $n_{D}=2^{n_{S}}$. From this, the total number of wires $N_{W}$ can be found with respect to the number of segments $n_{worm}$ (note the two extra wires to supply power. In the main schematic of the full design (Figure A5), these two wires are symbolised by the 5 V and GND labels).

$$N_{W}=\log_{2}n_{D}+n_{worm}+2$$

To implement the sensors along with the valves and one free port for expandability would mean eight devices per segment. An applicable component here would be the CD74HCT40XX series [54] of 3–8 multiplexer/demultiplexers. As such, a four-segment PALAM robot like the one designed would require just nine wires. A 20-segment-long robot would require just 25 wires, despite having over 200 individual control devices.

Another major advantage of multiplexers is the increased modularity and manufacturability. Thanks to the very small footprint of the electronics, the multiplexer, valve switching circuit, temperature sensors, and connectors for the linear potentiometers can all be mounted onto a single printed circuit board (PCB), which can be embedded in the segment. With the select and power rails (i.e., five common wires that run through the entire robot), the PCBs in the segments can be daisy-chained with a single additional wire needed for each segment. With today’s PCB manufacturing/soldering technologies using stencils and reflow ovens, this makes manufacturing many segments very easy and cost-effective.

#### 8.3.3. Feedback Algorithm

**Figure 19:**
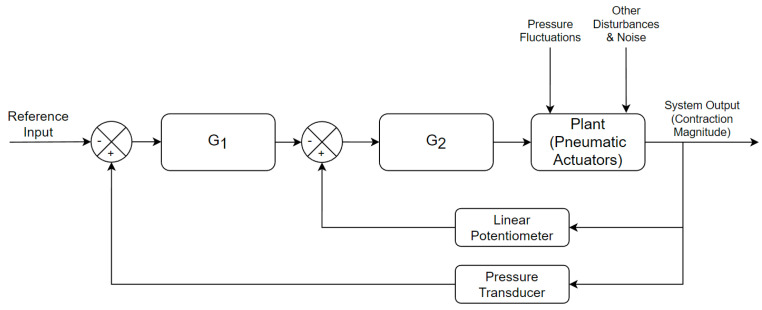
Feedback loop diagram of the system of one PAM.

Following the preliminary design of the prototype, a simple PID controller idea was devised. Figure 19 is a simplified block diagram of the system for an individual PAM. It consists of two controllers to correct the output for the feedback of two sensors. $G_{1}$ corrects the output depending on pressure fluctuations, allowing for longer valve opening times when the pressure is lower and vice versa. $G_{2}$ handles the actual position of the PAM by allowing for the valve to open and close according to the position feedback. It is important to note that the control input to the plant can only have two values; open and closed. The feedback therefore relies on the actuation time to achieve the desired system output.

The final design would have one feedback loop system for each actuator. In this configuration, the main motion algorithm sends a PAM position reference input to each feedback controller. The controller sends a message back to the main algorithm when the desired position has been reached. The algorithm can then move on when all desired PAM positions have been reached. This entire algorithm would be implemented in software and programmed onto the MCU. Thus, it is worth noting the controllers in Figure 19 would be “virtual” and not physical devices.

## 9. Manufacture and Experimental

### 9.1. Actuation

With an established pneumatic system and PAM design, the function of the PAM ultimately depends on material selection. For the core membrane of a GRACE PAM, a degree of elasticity and flexibility is necessary for unfolding (materials utilised in the GRACE proposal paper demonstrated elongation at break of 120%) [34]; however, excessively extending the membrane is unnecessary. For 1 mm thick membranes, materials with an elastic modulus of about 5 MPa were shown to ensure the desired contraction/elongation and generated forces. Additionally, friction between the PAM membrane and the contact interface must be maximised to support peristaltic motion.

The two main fabrication approaches considered for the GRACE PAMs were 3D-printed resin and cured material in a designed mould. In the GRACE proposal paper [34], PAMs were successfully 3D printed with a Form 2 printer and Flexible 80A resin filament (Formlabs Inc. Somerville, Massachusetts) [55]. In this way, Flexible 80A resin presented the most reliable option due to data from previous tests and simulations demonstrating no specific weaknesses in the membrane (with some force-limiting issues arising from the adhesion strength between layers). However, cured silicone, although time consuming, was the more feasible manufacturing option and crucially leads to high friction coefficients. While silicone has significantly increased flexibility compared with Flexible 80A resin [55] (the GRACE proposal paper assumes inextensive membranes [34]), it can be assumed that the membrane will not significantly stretch until the PAMs are fully inflated, at which point air flow will be stopped. The specific casting silicone utilised in the following manufacturing process was selected due to its accessibility, affordability, ease of use, and short lead time. The properties of materials considered in the selection process for Grace PAMs are provided in Table 2.

**Feasibility Checks**

After selecting a moulding approach and cured silicone for the PAM membrane, the suitability was verified with the MATLAB code. The range of suitable operating pressures for a silicone PAM were identified as between 1 and 6 kPa [34]. It was determined that the operating pressures equal to or below 5 kPa would be ideal, to mitigate risks of failure.

### 9.2. Segment Plates

With the design of the segment plates established, a rigid and durable material was needed for manufacturing. Three-dimensional printing was used to produce the plates in 3 separate parts, as this was identified as a reliable, accessible manufacturing method. The friction between the segment plates and the contact interface should be minimised to enable peristaltic motion. Graphite Aerosol Conductive spray was considered as an accessible option for ensuring this, if required following testing [57].

### 9.3. Manufacturing the PAMs

A mould casting process for silicone was selected for the PAMs. The manufacturing process for the 12 PAMs was developed and optimised, with multiple rounds, iterations, and experiments completed, leading to the finalised streamlined process of 5-day duration.

**Process Research and Considerations**

Before starting the manufacturing process, research was conducted into existing methods developed for mould casting with silicone. Woojun Jung et al. produced a micro-actuator [58] from Polydimethylsiloxane (PDMS) silicone using 3D-printed moulds. The moulds were printed in an acetone-soluble material (for easy removal), on a 3Z STUDIO printer and trialled at various print layer heights, ranging from 0.0064 to 0.0254 mm. A vacuum chamber was utilised for degassing the silicone after casting [58]. Similarly, Yongha Hwang et al. also fabricated an actuator [59] from PDMS silicone and a 3D printed mould, utilising an Objet24 printer with a 0.028 mm layer height and a vacuum desiccator for degassing. The moulds were printed as 1 mm thick cast shells filled with brittle support material to make them more pliant and therefore conducive to simple removal from the PDMS actuator after curing [59]. These examples suggest that 3D printing is an effective method for producing moulds, and that minimal layer height is desirable. The accessible printers within the current resource constraints have a minimum layer height of 0.05 mm, but it is believed that a layer height of up to 0.1 mm should be acceptable due to the comparatively reduced requirements for this assembly of the PALAM robot (the Jung and Hwang actuators are 1.2 mm and 15 mm in length before actuation, compared with the 40 mm PAM design). The mould removal techniques in these two examples were unfeasibly expensive and we therefore concluded that we would use PLA for the inner mould, to be melted for removal at 170 °C (melting temperatures of PLA are between 145 and 175 °C [49], while the maximum service temperature of silicone is 250 °C [49]). To ensure the success of this method, a test strip of cured silicon was heated to this temperature.

**Final Manufacturing Process**

**Figure 20:**
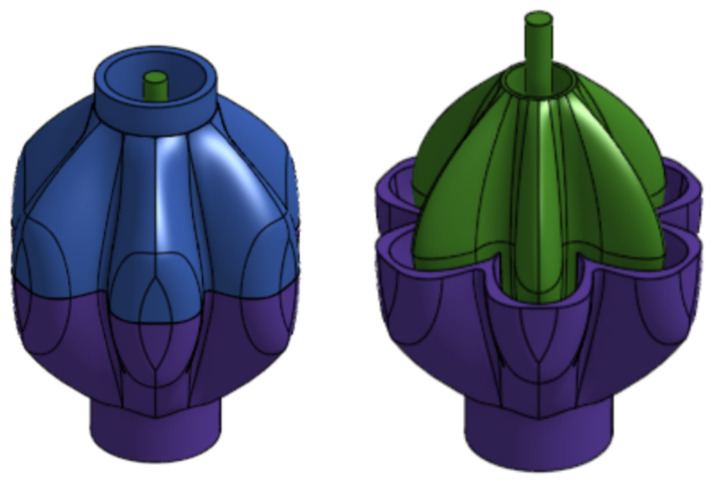
CAD model of final mould design.

Three different manufacturing processes and mould designs were trailed for the PAMs with numerous improvements being made to the design and manufacturing process each time. (Details and photos of each trial are included in Appendix C.1 and Appendix C.2). The final mould design, shown in Figure 20, was created on Onshape from an extruded box, from which the PAM model was subtracted. The PAM model had a built-in thickness, so the subtract function was used on the extruded box to divide it into two parts; one for the inner mould and one for the outer mould. The outer mould was then divided into two parts along the horizontal plane (using a spilt function) for easy access after curing and then sealed along the bottom. A small extrusion was added to allow the inner mould to lock into place. Then, 0.4 mm of clearance was added to account for the printing tolerances. The top half of the mould was left with a hole for the silicone to be poured into.

The moulds were 3D-printed on a Prusa Mini with a 0.1 mm later height and sprayed with a mould release spray. Then, the silicone was mixed for casting (silicone and curing agent were mixed in a 1:1 ratio), left to settle for around half an hour, and then poured slowly from a height into the mould. To remove air bubbles from the mixture a vibration table was used. After 24 h, the outer moulds were removed and the inner mould was then melted from the silicone PAM. During and after each manufacturing process, quality tests were carried out to ensure that the PAMs were suitable. The PAMs were inflated underwater to identify any weaknesses in the membrane. The finalised streamlined process of 3D printing moulds; mixing, casting, and setting the silicone; the removal of the outer moulds and melting of the inner moulds; and finally quality testing the moulds had a 5-day total duration.

### 9.4. Full Assembly

**Figure 21:**
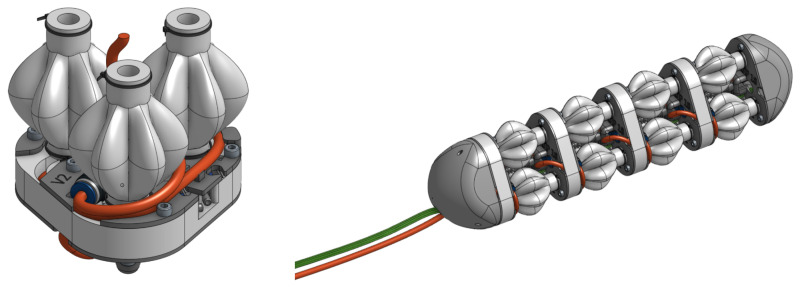
CAD of the final assembly.

The plates were 3D-printed in three separate parts, and assembled as shown in Figure 21, before being bolted together. The electronics was implemented as outlined in Figure 18.

### 9.5. Risks in the Manufacturing Process

The initial intended manufacturing approach for the PAMs closely followed the manufacturing method implemented in the GRACE proposal paper [34]. The GRACE proposal paper demonstrated that these PAMs performed well under a variety of forces and elongations, so this manufacturing method presented a low-risk option. However, due to budget and resource constraints, this was not a viable option [55]. Instead, the manufacturing process was reconsidered and ultimately mould casting for silicone was implemented. In order to mitigate the associated risks, initial research was carried out, before multiple iterations of the manufacturing process were completed. Each process involved experiments and improvements, as outlined in Appendix C.1.

The removal of the inner moulds after silicone casting was identified as a resource-intensive and high-risk stage in the manufacturing process for damaging the PAMs. Alternatives were considered, including 3D printing moulds from water-soluble PVA [60] and acetone-soluble ABS [61]. The ABS process was trialled and identified as an appropriate fallback due to its lower cost.

#### 9.5.1. McKibben PAM

The McKibben PAM [32,33,62] was considered as an alternative option to the GRACE PAM. Materials considered for the McKibben PAM include Nylon fibre, silk, and steel mesh [32] for the braided mesh and latex or silicone for the inner tube, as shown in Table 3. A form of Latex, likely natural latex, would have been selected for use of the inner tube McKibben PAM.

#### 9.5.2. Pleated PAM

**Figure 22:**
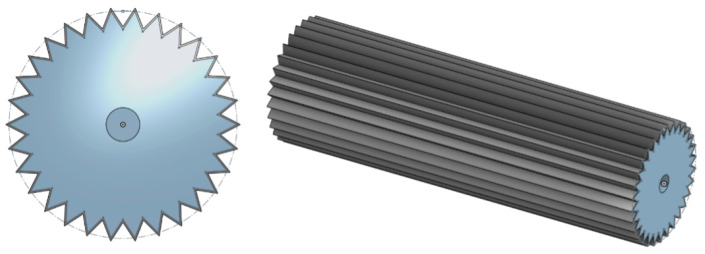
CAD model of a considered pleated PAM design.

The pleated PAM was considered as an alternative option to the GRACE PAM. A design was created for its implementation, based on successful existing pleated PAM designs [32,33,62]. The design, modelled as shown in Figure 22, consists of a 30-pleat membrane held between two end plates. The membrane is coated and sealed with a lining in order to achieve airtightness and ensure the pleats keep their original shape when deflating.

The materials considered for the membrane are shown in Table 4. Membrane materials need to have elastic behaviour and good fatigue resistance, due to the possibly sharp bends at the fold lines and the points of membrane fixing [33]. For the sealing of the membrane, a polypropylene lining was considered, glued to the fabric by a rubber resin adhesive [33]. The end plates could have been laser-cut acrylic or 3D printed with PLA.

Notably, manufacturing the pleated PAM would have been costly and difficult to ensure as the pleats functioned throughout operation, as well as achieving airtightness. However, it provided a viable, albeit less desirable, fallback option from the GRACE PAM.

### 9.6. Ensuring Peristaltic Motion

It is important to ensure both the rigidity of the PAMs, as well as their on demand deflation (and elongation). The potential challenges arising from the compliance of soft robots include the risk of deformation under external mechanical loads (such as structure collapse during rescue missions), with the potential for failure [21]. In the application of search and rescue, external loads could include structure collapse or debris impact. A finite element analysis was carried out with external point loads applied to the end plates and PAMs to determine the risk of this, detailed in Appendix C.3. The FEA confirmed that the plates would perform as required under loading.

## 10. Results and Discussion

### 10.1. Manufacturing Reliability and Quality

**Figure A13:**
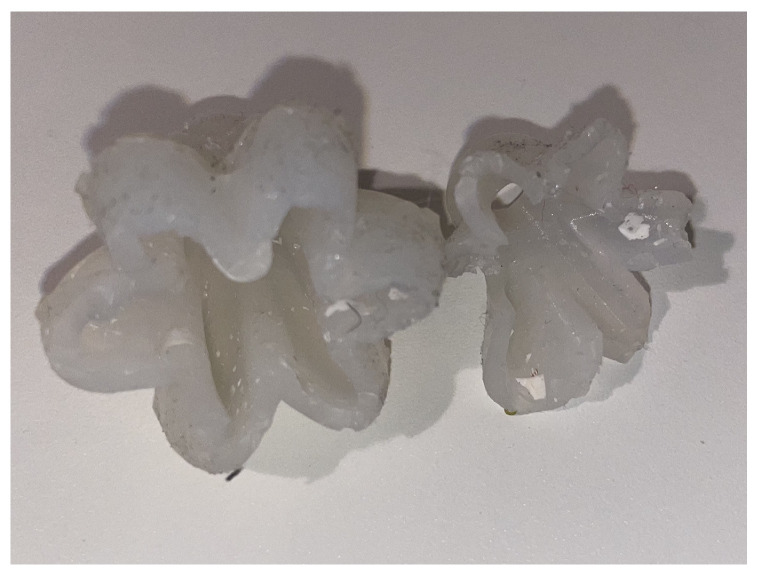
Photo of one of the ruptured PAMs. The rupture happened in about one quarter of the PAM during the demoulding process. Small remnants of the inner mould are visible.

A majority of pneumatic artificial muscles (PAMs) were successfully manufactured, proving the validity of mould pouring with silicone as a manufacturing method for producing GRACE PAMs. Of the 15 PAM manufacturing attempts, all moulds were cast successfully with no leaks of the uncured silicone rubber and all outer moulds were removed successfully without damage to the membrane. However, the removal of the inner mould was only successful for 12 out of 15 PAMs, due to issues with melting all PLA from the inner mould out of the PAMs within feasible time periods, plausibly due to surface tension keeping the viscous liquid stuck to the membrane. Sharp edges in the fused PLA ruptured the membrane of three PAMs, as shown in Figure A13.

**Figure A14:**
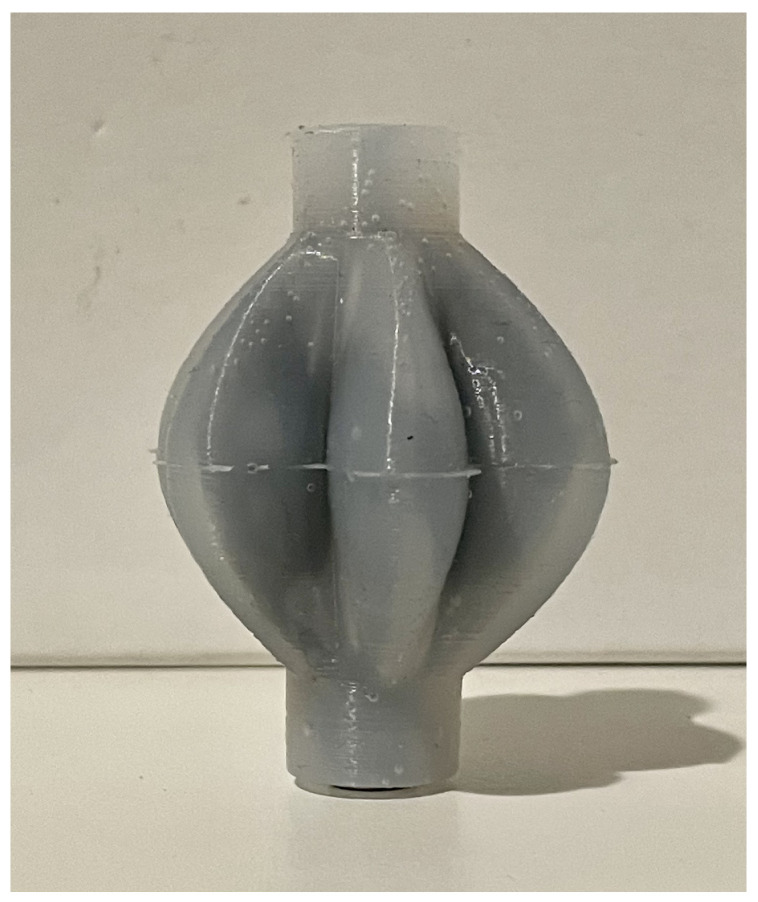
Photo of PAM before inner mould removal. The small cavities are noticeable near the top of the photo.

Tests were carried out on the remaining 12 PAMs to ensure uniform manufacturing and sufficient quality. Air tightness was tested through simple underwater inflation tests. Nine PAMs were sufficiently airtight, and three had small pin-hole leaks, due to bubbles trapped in the silicone during the curing process. As uncured silicone rubber adheres to its cured form, these holes were patched and the PAMs rendered airtight. The quality of the membranes were only further inspected visually. The membrane thicknesses were uniform, proving the success of the locking method at the bottom of the moulds at keeping the inner mould centred, and that the uncured rubber evenly filled all gaps. However, across all PAMs, air-bubble-induced cavities were found, as shown in Figure A14, increasing towards the top of the PAM. It is likely that the bubbles were trapped at the curvature of the top of the outer mould, and between the 3D-printed layer ridges. Overall, the casting and testing phases proved successful, with 9 out of 15 PAMs passing all initial tests and 3 passing after minimal repairs. The final success rate was thus of 80%.

The weight and dimensions were found to be uniform across the 12 PAMs selected for the robot, with an average of 17 g and 40 mm deflated length (see Appendix D.1 for dataset). The uniformity of the weights and dimensions show that the tolerances of the 3D-printed moulds were well within an acceptable limit. Notably, however, these weights are greater than the comparable weights for GRACE PAMs manufactured with flexible resins (recorded weight of 6 g of equal height, 40 mm) [34] and so this manufacturing method may lead to reduced force-to-weight ratios, as discussed below.

### 10.2. Actuation Performance

The operational performance of individual PAMs, isolated from the full assembly, was tested. Under no-load conditions, the maximum inflation was reached at an operating pressure of around 5 kPa. Beyond this pressure, the PAMs began to exhibit a balloon-effect, expanding in all directions and elongating rather than contracting. In comparison to the GRACE proposal paper [34], the PAMs of equal dimensions and 1 mm thickness perform at similarly low pressures (in the order of 10 s of KPa), while this can be increased for increasing stiffness or thickness of the membrane material.

**Figure 23:**
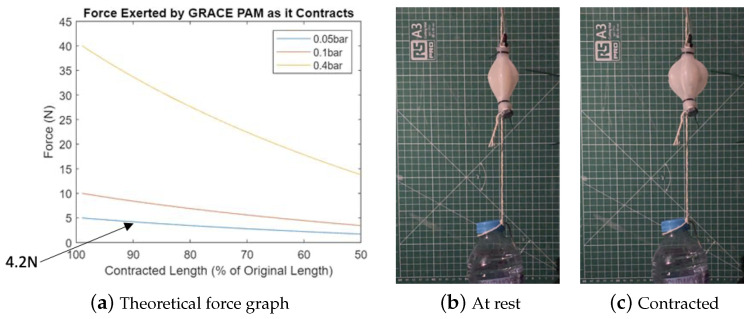
PAM contraction theory and tests. The graph in Figure (**a**) is for a GRACE PAM of deflated length 0.04 m and end diameter 0.016 m operating at 5 kPA, 10 kPa and 40 kPA.

Force–contraction tests were carried out at constant forces, limited to 5 N in order to avoid destructive testing. These values were sufficient, as they exceeded sufficient forces for the operation of the PALAM robot. Figure 23a–c show the theoretical and experimental contractions at 5 N, generated by a PAM of manufactured dimensions: deflated length 0.04 m and end diameter 0.016 m. Theoretical values are 10% contraction (90% of original length) at 4.2 N. Experimentally, a constant force of 5N was maintained, while the average elongation was found to be 5 mm (12.5% contraction, 87.5% of original length) at the working pressure of 5 kPa.

The contraction forces exerted by the silicone PAMs closely match those predicted by theory. The Chou and Hannaford model (see Equation (3)) was used to produce theoretical values, and is known to overestimate forces compared with experimental values. This suggests that the PAMs produced for the PALAM robot have reduced force capabilities for similar pressures in comparison with previous pleated PAMs developed [32]. Additionally, the maximum contraction appears to be limited to 10%, while the GRACE proposal paper [34] suggests a possible contraction of up to 50% with the used geometry. This is likely due to the hyper-elastic nature of silicone rubber, which causes the membrane to excessively stretch the entire PAM outwards (thus causing premature expansion in all directions) before the pressure is able to rise enough to allow further contraction.

The achieved force-to-weight ratio reaches 300%, although clearly higher values could be achieved if testing approached destructive testing. This can be compared with the force-to-weight ratios achieved by the GRACE PAM proposal paper: utilising 3D-printed flexible 80A resin, a ratio of 167% is achieved, while 3D Printed Flexfill TPU 98A achieves 1000% [34].

The success of this unprecedented method for manufacturing GRACE PAMs confirms the theory that the GRACE geometric design allows for production from a range of materials. Furthermore, this adds to the existing knowledge in the field of PAMs, providing an accessible and affordable manufacturing option (utilising Prusa Mini 3D-printers and PLA Filament, rather than Form 3 3D-printers and 80A flexible resin). In addition, silicone provides major advantages in terms of chemical inertness and heat resistance. There are hence clear potential applications for these PAMs in various PALAM robot designs. For instance, with high-temperature grade electronics and metal segment plates, the robot may be used for search and rescue in fires. Another application is endoscopy, using surgical grade silicone rubber PAMs and inert stainless steel segment plates.

### 10.3. Reliability and Build Quality of the Fully Assembled PALAM Robot

Following the full assembly of the PALAM robot, preliminary testing was carried out with a simple algorithm to individually activate each valve in sequence. With minor tweaks to the switching circuitry (such as increasing pull-up resistor values from 1kΩ to 10kΩ), the valves operated successfully, each of them fully activating and closing with negligible delay.

Challenges arose in ensuring the impermeability of seals; the tubes were difficult to correctly and fully insert into the manifold’s push-fittings, especially in the full assembly. In fact, the first pressurisation test proved to be a failure, with 4–5 fittings leaking, although this was easily fixed through disassembly, testing for leaks at each manifold and correcting fittings. Once reassembled, the PALAM segments were able to successfully contract and elongate longitudinally (as they were inflated and deflated), reducing, and increasing the length of the entire robot. The segments were also able to expand and contract radially (as they were inflated and deflated). This allowed them anchor to the contact interface while fully inflated and lift off the interface, held up by the segment plates, while deflated. This validated our initial motion modelling.

Before performing timed tests, the preliminary testing of peristaltic motion was carried out by means of the manual pressurisation of the worm using a hand pump. The initial results were not promising, as the PAM contractions seemed to be insufficient to ensuring reliable displacement of the robot. While the hand pump did achieve a moderate inlet pressure of approximately 20 kPa, this dropped significantly at each PAM contraction. Thus, a higher pump flow-rate would be needed. Furthermore, it was found that, while leaks were mostly sealed at low pressures of up to approximately 5 kPa, air was still leaking from the fittings and PAMs at higher pressures. It was found that, due to friction losses from the pipe, the working pressure in the first segments needed to be higher. This ultimately resulted in a constant supply leak, exacerbating the low flow-rate issues.

## 11. Motion of the Fully Assembled PALAM Robot

### 11.1. Peristaltic Motion

#### 11.1.1. Implementation and Test Setup

Testing was achieved by programming the first motion model into a timed sequence. The various timings between segment actuations were set-up as variables at the start of the C code and roughly implemented according to the inflation time of the PAMs. However, it was immediately noted that the inflation of the PAMs was significantly quicker in the final segment (closest to the supply), due to the lower pressure compared to the first segments. While this would be fixed by a closed-loop feedback loop, here, the timings of the second phase (the deflation phase) were simply reduced. This was also found to improve efficiency, as deflation was always quicker.

The motion of the PALAM robot was started by plugging the programmed Arduino Due (the micro-processor) into a 5 V supply using a USB cable. An electric pump with an air cannister as a buffer to stabilise the supply pressure were connected to the supply line to supply the PALAM robot with pressure. Once the Arduino started operating and the valves started activating in sequence, the pump was turned on. As expected, the PAMs activated successfully and in sequence with minimal problems. Movement was slow but still clearly visible. The next step was to quantify the velocity of the worm and explore methods to optimise it. This was achieved using a gridded background and timing the movement.

#### 11.1.2. Timings and Results

**Figure 24:**
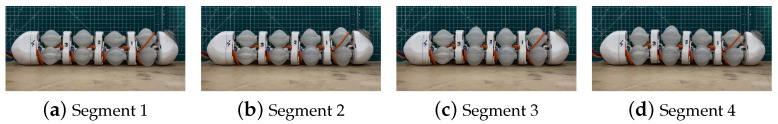
Steps of peristaltic motion contractions shown on the PALAM robot (see Figure 25 for elongated state).

The PALAM robot was tested with various timing sequences, Figure 24. The interface for the robot was a work-bench made of bare wood (no coatings). Each test lasted one minute, after which the total displacement was recorded. The results are detailed in Table 5.

### 11.2. Testing with Bristles

**Figure A15:**
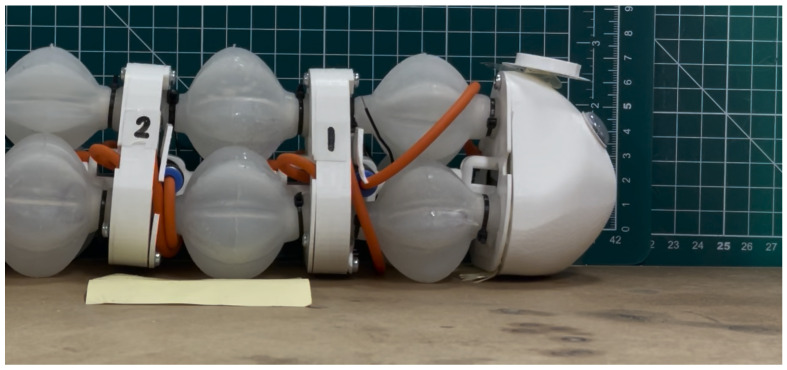
Photo of setae (bristles) added to the head of the worm.

A brief experimentation phase made use of small bristles at the bottom of the PAM segments, shown in Figure A15, as an alternative anchoring method to friction at the PAMs, following the second motion model. The introduction of bristles of various lengths appeared to worsen the total velocity on the smooth test surfaces experimented on. However, brief testing suggested it could improve anchoring on rough surfaces, onto which the PAMs may not be able to anchor sufficiently.

Ultimately, setae offer better anchoring to rough and slippery surfaces. We believe the PAM friction will be inadequate on rough, wet, muddy, or dusty terrains, and setae will be vital; however, this will also force the robot to unidirectional motion only, as opposed to its current bidirectional design following the first motion model. This may be problematic for tight space navigation, where backwards motion could be exploited, while for other applications, setae may be advantageous. Further future testing is required to confirm the viability of this anchoring technique. Alternative methods for ensuring unidirectional movement include outer shell designs that provide low friction moving forward but high friction moving sideways or backwards [7].

### 11.3. Achieving Three Degrees of Freedom

The PALAM robot’s segments were designed with three PAMs to allow for a full range of motion of each segment. While testing and tuning was focused on the hypothesis of peristaltic motion, a few tests were conducted regarding the theory that three degrees of freedom could be achieved. This was simply achieved by repeating the previous tests but drastically shortening the actuation period of one of the PAMs, in this case, the bottom right PAM in the direction of travel, in order to cause the robot to turn slightly to the left.

During testing, the robot continued to move forward, albeit at a slightly reduce speed. This confirmed that even with one sub-actuated PAM, peristaltic locomotion could be maintained. The entire structure also noticeably deflected leftwards, confirming the theory that 3-PAM segments allows for rotation. The deflection was minimal, causing the worm to very slightly turn leftwards, with the heading changing by about 1–5 deg in one minute. However, it is believed that having more segments would increase the deflection, although further testing would be needed to determine this.

The first model of peristaltic motion has proven to be a valid locomotion technique for the PALAM robot. The tests showed that the cured silicone rubber has a sufficiently high friction coefficient and 3D-printed PLA has a sufficiently low friction coefficient with the interfaces tested to produce peristaltic motion. Motion velocity was fairly constant despite various timings, and the maximum velocity reached was 0.3. While certain timings showed a slight improvement, these must be further tested to ensure they are not due to measurement errors and tolerances within the pneumatic system.

A first likely cause for the slow movement is the low flow-rate in the tubes (and thus slow PAM contraction), highlighted by the much faster deflation rate of the PAMs (due to the air being ejected into the atmosphere through the valve). However, the deflation rate is still non-negligible, and it is likely that with improved tubing it would become the next “bottleneck” of the system. Thus, the maximum actuation time would reach approx. 100 ms down from 400 ms, which in theory would only be increasing the total velocity by 3–4 fold.

Another potential cause is the hyper-elasticity of the PAM membrane. First, it was shown that this causes a low contraction ratio, which is directly proportional to velocity. If another material were used and the theoretical contractions of 30–40% were achieved, the velocity could be increased 3- to 4-fold. Second, the elasticity of the PAMs causes poor anchoring. This is noticeable in the locomotion test videos (cf. Electronic Supplementary Materials), where despite the friction being adequate, the PAM “wobbles”, causing the segments plates to still be pulled towards the other contraction PAMs. The “wobble” thus negates part of the actuation. Third, the PAMs were not perfectly rigid when elongated, acting like springs when pushed forwards by elongating segments. This negated part of the elongation process.

### 11.4. Implications

Depending on applications, a balance must be found between the velocity and material properties of the PALAM robot. It is believed that using a less elastic PAM with larger tubing would increase the final speed more than 10 fold, with speeds of over 3 not unreasonable for the current robot size. However, this speed is still significantly slower than other robot locomotion techniques, such as driven wheels. This suggests that the application scope of a PALAM robot may be limited in many aspects; if there is enough available space, it would likely always be better to use a wheeled robot. Hysteresis is another factor of importance to consider. Since the peristaltic motion of the PALAM robot is unidirectional, dry friction, causing hysteresis, presents itself between the PAM and the surface along which it moves in the axis of the robot. Hysteresis is therefore a function of PAM expansion and contraction as the robot moves forward. As such, the motivity of the contacting surface determines the extent of hysteresis. As discussed by Dardeen and co-workers [32], a method by which means hysteresis can be minimised, even eliminated, is through the use of folding/unfolding actuators, which have sufficient instability to enable tilt rather to be bound by frictive drag when moving, as is the case with the PAMs.

However, the PALAM robot still shows potential in many search operations where a soft-bodied, flexible robot is needed. Its ease of manufacturing and simple locomotion system offer good reliability and less points of failure, making it an attractive option where dust, water, heat and chemicals could quickly degrade a more complex mechanical system. For example, only minor modifications would be needed for the robot to be fully water-proof and able to navigate in pipelines. Furthermore, the high forces of the PAMs can prove useful in many cases where loads must be carried or debris freed. This goes beyond the scope of this paper and the robot was designed without taking full advantage of the forces that the PAMs can exert. However, by improving anchoring, the segment plate design, and the pneumatic system, the PAMs at the core of the PALAM robot can be fully exploited to provide much more force than other locomotion systems. This could be useful to allow a relatively small robot carry significant loads, such as removing debris or moving people in search and rescue applications. The high force-to-weight and force-to-volume ratio can also be useful if the form-factor of the robot is decreased.

In this work, our major point of innovation is in the use of PAMs in conjunction with separation plates to enable both peristaltic and nonlinear motion across the several individual “modules” of a worm-inspired robot. To date, robotic peristalsis using soft pneumatically actuated muscles have only been evidenced using single structures, e.g.,: heat-cooled single soft-fibre artificial muscles [63], and single-concertina-based soft structures [64]. While these have also shown peristaltic motion, they are single actuated structures. Our robot is an enabler of linear and nonlinear motion, and comprises four linked modules, each of which is individually controllable.

## 12. Conclusions

Our original hypothesis was that peristaltic motion can be mimicked by a soft-bodied modular robot, consisting of four repeating segments that are each individually pneumatically actuated by three artificial muscles. The PALAM robot achieved the successful manufacturing of such a soft-bodied modular robot, consisting of four such segments. These segments are able to successfully contract and elongate, as well as anchor themselves to a contact interface, replicating the biological sets of muscles of earthworms. The PALAM robot is thus able to achieve peristaltic motion in either direction. The soft-bodied, flexible PALAM robot shows potential in many search and rescue missions, such as navigating unstable environments and inaccessible caves. The simple locomotion system offers reliability, compared to more complex mechanical systems, and its compliance and lightweight design lends itself to safe interactions with the environment and human users. Lastly, the high force-to-weight ratios achieved by the actuation system show potential for the PALAM robot as a successful carrier, e.g., of search cameras and search and rescue equipment. Finally, a new and more accessible manufacturing method for pneumatic artificial muscles (PAMs) was successfully developed, optimised, and validated.

## Figures and Tables

**Figure 25:**
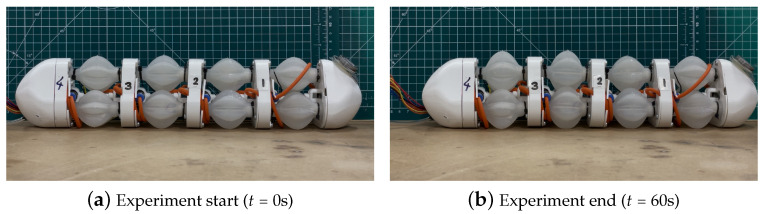
Photos of the start and the end of the experiment which gave the fastest speed (40 mm in 60 s). Also refer to Electronic Supplementary Materials for the video showing peristaltic motion. **Note:** The reference point is the thick line of the grid in at the left of segment plate 2. Each grid division measures 10 mm.

**Table 1:** Geometrical parameters resulting from the optimisation implemented by the geometrical model for the three main GRACE designs: GRACE-C, GRACE-E, GRACE-A [34]. Note that parameter $b_{1}$ in grey is not an independent variable. The parameters of interest here are from the shaded maximum contraction column.

	GRACE-C	GRACE-E	GRACE-A
**Parameter**	**Max Contraction**	**Max Elongation**	**Antagonistic**
$a_{1}$	0.48	0.1	0.24
$b_{1}$	0.17	0.26	0.29
$a_{2}$	0.16	0.06	0.09
$b_{2}$	0.11	0.22	0.13
$R_{2}$	0.53	0.9	0.8

**Table 2:** Material properties of considered PAM materials [34,49,55,56].

Material Properties	Post-Cured Flexible 80A Resin	Post-Cured Standard Clear V4 Resin	Silicone Elastomers (General)
Flexural modulus (bending) (GPa)	5	2.2	0.000005–1.90
Elongation at break (% strain)	120	6	270–600
Tensile strength (MPa)	8.9	65	7–11.5
Performance			
Accessibility/affordability			
Manufacturability			

**Table notes:** Note that friction coefficients are not included due to the inaccessibility of specific, consistent information suitable for direct comparison. *Performance:* Based on material properties, the PAM performance is likely to be achieved with specified material. *Accessibility/affordability:* Accessibility given the budget and resource constraints. Note that the materials available through the University are not counted towards the budget, and so score high on affordability. *Manufacturability:* Ease and feasibility of manufacturing a PAM from material given the 8 week timeline. 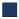 Low score—least desirable option. 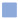 Medium. 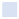 High—desirable option.

**Table 3:** Material properties of materials considered for McKibben PAM [37,49].

Material Properties	Silicone Elastomers (General)	Polyurethanes (General, Incl. Lycra, Spandex)
Yield strength (elastic limit) (MPa)	7–11.5	40–51
Elongation at break (% strain)	270–600	500–750
Young’s modulus (tens. or compression) (GPa)	0.005–0.05	0.0025–0.03
Tensile strength (MPa)	7–11.5	40–51
Fatigue strength at 10^7^ cycles (Mpa)	2.8–4.6	16–20.4
Performance		
Accessibility/affordability		
PAM manufacturability		

**Table notes:** Note that the same table notes apply as for Table 2. 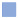 Medium. 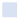 High—desirable option.

**Table 4:** PPAM [49].

Material Properties	Silk (Silkworm Silk) Fibre	Aramid Fibre (KEVLAR 49)	Aramid Fibre (KEVLAR 29)
Flexural modulus (bending) (GPa)	9.3–15	117–130	62–80
Yield strength (elastic limit) (MPa)	340–720	2250–27,500	2500–3000
Elongation at break (% strain)	18–35	2.4–2.6	2.5–4.4
Young’s modulus (tension or compression) (GPa)	5–25	125–135	62–80
Tensile strength (MPa)	340–720	2500–3000	2900–3600
Fracture toughness (MPa)	1–2	2–4	2–4
Accessibility/affordability			
PAM manufacturability			

**Table notes:** Note that the same table notes apply as for Table 2. 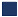 Low score—least desirable option.

**Table 5:** Peristaltic motion experiments results with various timings.

Timing Var.	Delay Explanation (All Delays in ms)	Exp1	Exp2	Exp3	Exp4
firstPAM	After segment 1 PAM activation	400	200	400	400
wormDelay1	Between PAM activations for segments 2–4 during contraction seq.	400	200	200	400
wormDelay2	Between PAM de-activations during elong. seq.	100	100	100	150
cycleDelay	Between cycles (in addition to a wormDelay1)	300	300	300	300
**Experiment Runtime (in s)**		60	60	60	60
**Distance Travelled (in mm)**		40	35	40	30
**Speed (in $\mathrm{mm}\cdot s^{-1}$)**		0.67	0.58	0.67	0.5

**Figure A1:**
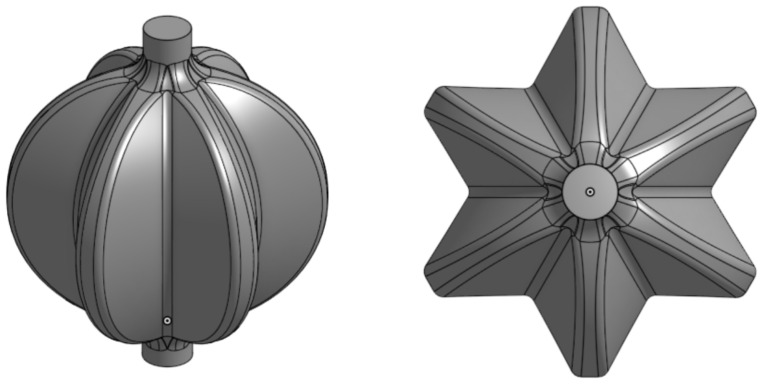
First prototype of the GRACE PAM.

**Figure A6:**
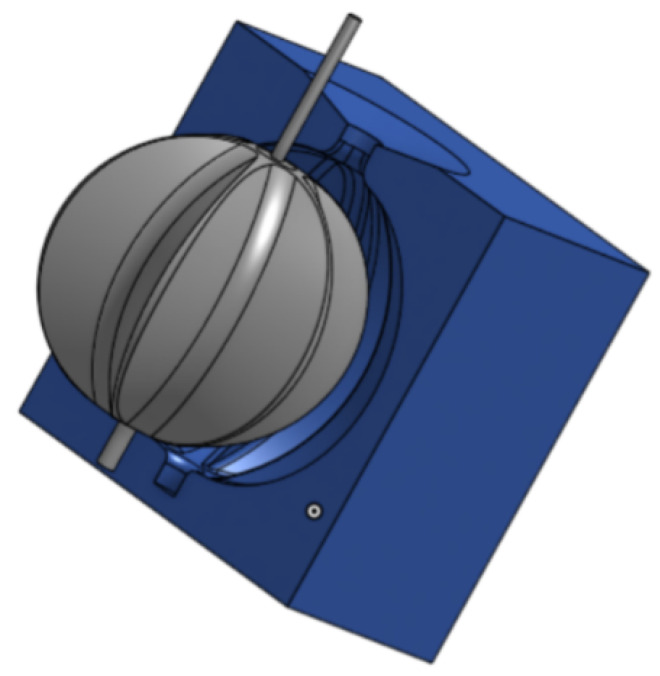
CAD model of initial mould design.

**Figure A7:**
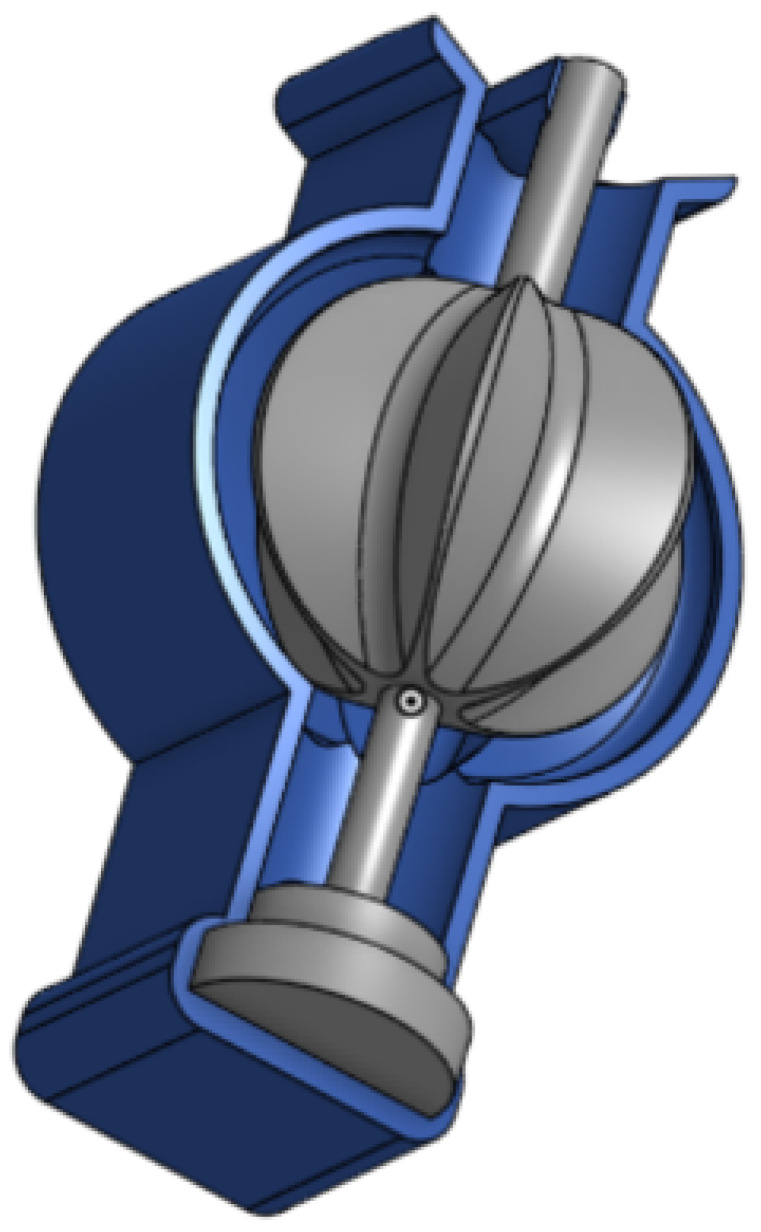
CAD model of a second version of the mould design.

**Figure A8:**
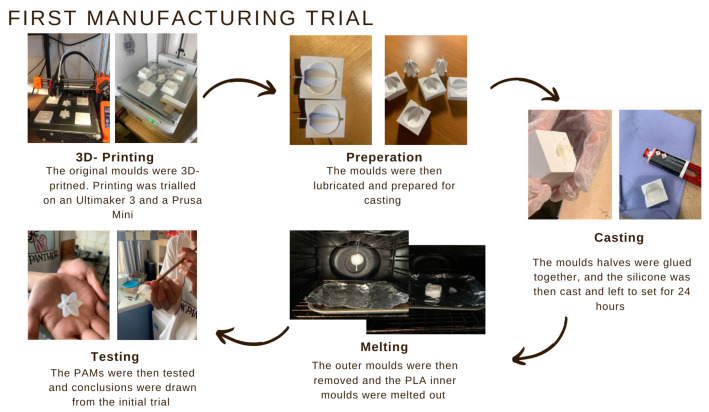
First trial of the manufacturing process.

**Figure A9:**
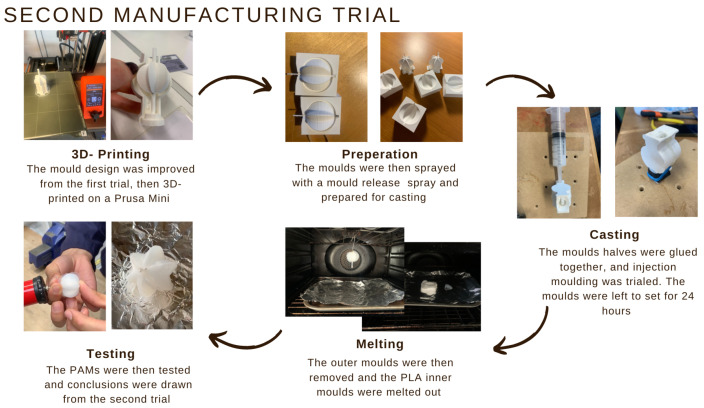
Second trial of the manufacturing process.

**Figure A10:**
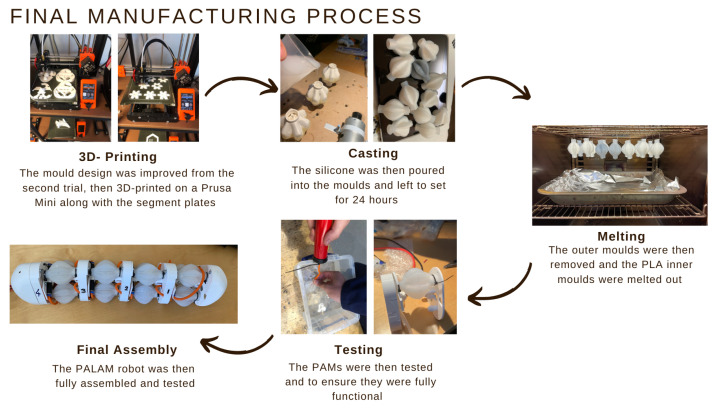
Final manufacturing process.

**Figure A11:**
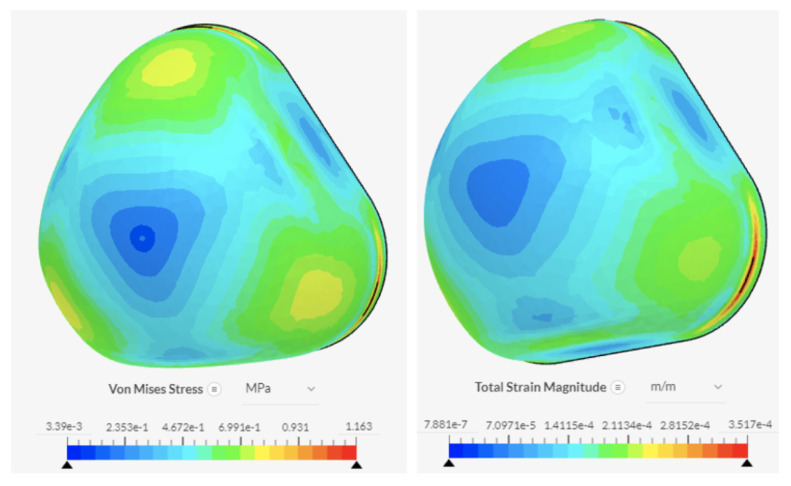
FEA of straight-on head-loading (**a**) Von Mises stress (**b**) total strain magnitude.

**Figure A12:**
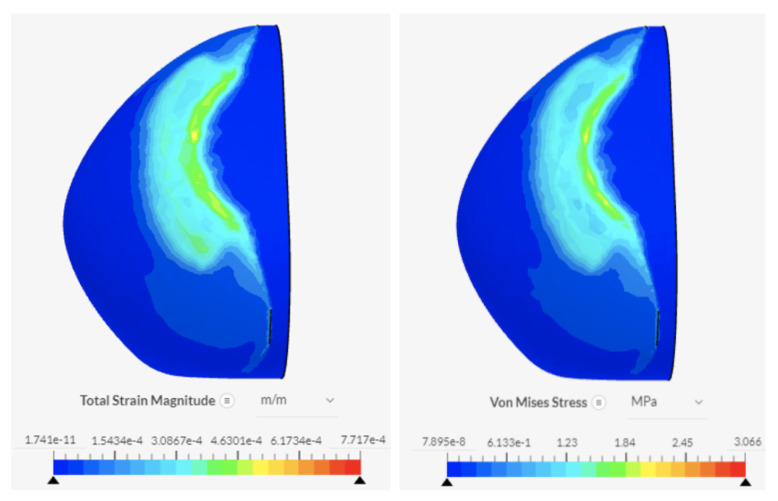
FEA of side-on head-loading (**a**) Von Mises stress (**b**) total strain magnitude.

## Data Availability

Data are available from the corresponding author upon request.

## Supplementary Materials

The following supporting information can be downloaded at: https://www.mdpi.com/article/10.3390/biomimetics9080447/s1, Matlab Code S1: Force of GRACE PAM; Matlab Code S2: Force of McKibben or pleated PAM; Matlab Code S3: Friction coefficient; Matlab Code S4: Parabolic curves; Video S1: timelapse video of segment assembly; Video S2: timelapse video of final assembly adjustments; Video S3: Video of PAM in action; Video S4: PALAM Robot in motion at ×2 speed.

## Abbreviations

The following abbreviations are used in this manuscript:

ABS	Acrylonitrile butadiene styrene
CAD	Computer-aided design
CCS	Centre cross-section
CNC	Computer numerical control
DIC	Digital image correlation
DoF	Degrees of freedom
EOM	Equations of motion
FEA	Finite element analysis
FBD	Free body diagram
GRACE	Geometry-based actuators that contract and elongate
GRACE (C,E,A)	GRACE contraction, elongation, and antagonistic
ID	Inner diameter
OD	Outer diameter
PALAM Robot	**P**neumatically **A**ctuated perista**L**itic **A**dvancing **M**odular Robot
PAM	Pneumatic artificial muscle
PDMS	Polydimethylsiloxane
PID controller	Proportional—integral—derivative controller
PLA	Polylactic acid
PP	Polypropylene
PPAM	Pleated pneumatic artificial muscle
PVA	Polyvinyl acetate
SLA	Stereolithography
TPV	Transportable pressure vessel
MCU	Micro-controller unit

## Appendix A. Design Theory

### Appendix A.1. Anchoring and Resistive Forces

To calculate maximum anchoring and resistive forces $F_{A}$ and $F_{R}$, a friction law was selected. The Coulomb friction model (see Equation (A1)) was used for multiple reasons. Despite a significant simplification (leading to errors of up to 20% [65]), the model has been deemed sufficient to model peristaltic motion in peer-reviewed papers [10,11]. Moreover, the Coulomb model is an efficient ‘standard model’ [10] used ubiquitously as a first-order approach across engineering and wider scientific fields. The general friction force Equation is as follows:

(A1) F=μN

where μ is the friction coefficient and *N* is the normal force.

The coefficient of friction can take the value of the static coefficient of friction, $\mu_{s}$ (where there is no velocity) or the dynamic coefficient of friction, $\mu_{d}$ (when velocity is greater than 0). Static coefficients produce higher friction forces and are utilised in this static model, as no movement is assumed in the case of Segment A, and the maximum resistive force acting on Segment B is needed.

Applying Equation (A1) to the anchoring force first (for which the case of static friction applies) gives:

(A2) $$F_{A_{max}}=\mu N=\mu_{1}W_{segment}$$

where $\mu_{1}$ is the friction coefficient and *N* is the normal force.

This calculation assumes that two PAMs in a segment will make contact with the interface, but that all three PAMs in a segment will make contact with each other, so that the normal force is the weight of all three PAMs.

The maximum resistive force experienced by any one segment will occur when Segment 1 is contracted, and Segments 2–4 are dragged. In this situation, the maximum resistive force on Segment 1 will be the friction force at three plates.

(A3) $$\begin{array}{c} F_{R_{max}}=3\left(\mu_{2}N_{plate}\right)+\mu_{2}N_{end}\\ \;\\ N_{plate}=\left(m_{plate}+3m_{PAM}\right)g\\ \;\\ N_{end}=\left(m_{end}+\frac{3}{2}m_{PAM}\right)g \end{array}$$

where $\mu_{2}$ is the static friction coefficient between the segment plates (or end plates—assumed same coefficient) and the interface. For simplification, this calculation assumes the plates each take the additional weight of one segment, (so that the normal force at a plate is the weight of one plate and one segment), except for the normal force, which takes the additional weight of half a segment (so that the normal force at a plate is the weight of one plate and half a segment).

### Appendix A.2. Theoretical Force Model Selection and Validation

Multiple models were considered for calculating the theoretical forces generated by PAMs. The first model was selected to enable the use of a single approach that can be adapted to estimating the force for a range of PAM types and materials.

1. The first mathematical model considered allows force to be calculated without any consideration of the detailed geometry of a PAM, using only the inlet pressure and state of inflation, as shown in Equation (A4). This model demonstrates that increasing the contraction leads to a reduction in generated force.
  (A4) $$F=\rho \frac{dV}{dL}$$
  where ρ is the pressure gauge in the PAM, dV is the change in the volume of air enclosed in the PAM and dL is the contraction, that is, the change in the PAM’s axial length compared with its maximum value at deflation [32,33,41,42]. Quasi-static conditions and negligible energy losses (including the energy required to deform or rearrange the membrane) are assumed [33,41]. This model was first proposed by Chou and Hannaford in a 1996 report on McKibben PAMs [42], derived from the net amount of work crossing the PAMs boundary [32]. It has since been applied and validated in wider-ranging research that has included alternative PAM types [32,41]. However, it should be noted that the neglected energy losses in the model lead to produced theoretical values that exceed the measured forces [32].
2. The second mathematical model considered, shown in Equation (A5) introduces $f_{t}$ a dimensionless function dependent only on a contraction rate, geometry, and material behaviour. This model applies to pleated PAMs. Experimental results agree extremely well with the values derived from the mathematical model [32,33,41].
  (A5) $$F=\rho L^{2}f_{t}$$
3. The third mathematical model considered, shown in Equation (A6), applies to McKibben PAMs and requires the weave angle, θ, of the braided mesh [32].
  (A6) $$F=\frac{\pi \;D_{max}^{2}\;\rho }{4}\left(3cos^{2}\theta -1\right)$$
  where $d_{max}$ is the PAM’s diameter at a braid angle of 90 degrees.
4. The fourth mathematical model considered, applied to pleated PAMs with fibres lining each the crease of each pleat, is shown in Equation (A7):
  (A7) F=nσs(1−2m)
  where *n* is the number of pleats, σ is the tensile stress along the fibres, *s* is cross-section of the fibres, and *m* is an integral parameter that ranges from 0 to 0.5 in (detailed derivation and solution can be found in the model’s proposal paper [41]). The resulting theoretical solution is almost identical to that of the Chou and Hannaford model [41], and so one can be used to check the correct implementation of the other.

### Appendix A.3. Volume Models

Once the Chou and Hannford model was selected, identifying an approach to estimating dV was key. Previously published simplified models for estimating PAM volumes have included: utilising the assumption that the PAM volume only depends on length [42] (this cannot be applied in a context where such an emphasis is placed on radial expansion (for example peristaltic motion)); the approach of modelling portions of PAMs with simplistic shapes [42] such as cylinders; and the widely used simplified model of the Pleated PAM as having a circular cross section (instead of a pleated cross-section) of diameter equal to the average of the outer and inner diameter of the pleats [41]. The latter two could be utilised here, and hence, we employed the following models for estimating dV:

i The deflated McKibben PAM was approximated with a cylindrical model. The inflated volume is modelled as two adjoining truncated cones of increasing central diameter (starting at the cylinder’s diameter), while the axial length of each cone is decreased and the diagonal lengths are held at constant length. The increasing volume due to the stretch of the McKibben PAM during operation is not taken into account.
ii The model of the Pleated PAM was approximated with a circular cross-section (instead of pleated cross section) of diameter equal to the average of the outer and inner diameter of the pleats. In this way, the Pleated PAM was similar to the McKibben as a cylinder (due to the end plates) when deflated, and as two adjoining truncated cones of increasing central diameter (starting at the cylinder’s diameter) during inflation. Again, the axial length of each cone is decreased and the diagonal lengths are held at constant length. The increasing volume due to the rearranging membrane (unfolding pleats) is not taken into account.
iii The deflated GRACE PAM was modelled as two adjoining truncated cones (due to their reduced relative end plate areas), again using a simplification to average the diameter of the pleats. To model increasing contraction, the central diameter of the two adjoining truncated cones can be increased while the axial length of each cone is decreased and the diagonal lengths are held at constant length (as there is no substantial stretch of a GRACE PAM during correct operation). The unfolding of the pleats during operation is not taken into account.

Notably, these models do not account for the stretch of McKibben PAM or the unfolding pleats of Pleated and GRACE PAMs (both of which will contribute to the change in volume dV) and so ultimately lead to an underestimation of the change in volume dV. Despite this, these models remain useful for feasibility calculations. As Chou and Hannaford’s method already overestimates force generated, an overestimation of dV can help to compensate. Moreover, an underestimation of the force generated would be useful for an estimate, to ensure the force generated will be excessively sufficient for peristaltic motion to be achieved.

## Appendix B. Design Analyses

### Appendix B.1. First PAM Prototype

To verify the viability and manufacturability of the PAM design, a first version was developed by visually replicating the GRACE design from the GRACE proposal paper [34]. This was achieved using the sweep feature to remove triangular section from a sphere, which created the six pleats. Fillets were then used to smooth the edges, the shell feature used to make the PAM hollow and the mounting holes added by extruding a circular sketch on either side of the PAM. This design was then used to prove the viability of the manufacturing process.

### Appendix B.3. FEA

To conduct the FEA, we used the following material properties inputs:

**Table A1:** Material properties (information sourced from Granta Edupack [49]).

Component(s)	Material	Behaviour	Young’s Modulus (GPa)	Poisson’s Ratio	Density (kg/m^3^)	Elastic Limit (MPa)
**PAMs**	Silicone Rubber	Hyper elastic	0.02	0.48	2000	2
**Cable ties**	Nylon 6,6	Linear elastic	1.8	0.4	1150	1.8
**Solenoid valves**	Polypropylene (PP)	Linear elastic	1	0.45	900	1
**Segment plates**	Polylactic acid (PLA)	Linear elastic	3.5	0.36	1250	3.5

**Table A2:** Mass calculations [49].

Component(s)	Volume (mm^3^)	Density (kg/m^3^)	Mass of Each Component (g)	Total Number of Pieces in Assembly	Total Mass (g)
**PAMs**	14,955	2000	29.91	12	358.91
**Solenoid valves**	-	-	25.60	12	307.20
**Segment plates**	9804 (assuming 15% infill of 3D print) [6]	1250	12.25	4	49.00

The static friction coefficient used for the segment plates on the contact interface was 0.492, assuming the use of PLA [66].

### Appendix B.4. Equations of Motion Calculations

Using Lagrange’s equation, $\frac{d}{dt}\left(\frac{\partial T}{\partial {\dot{q}}_{i}}\right)-\frac{d}{dt}\left(\frac{\partial T}{\partial {\dot{q}}_{i}}\right)+\frac{\partial V}{\partial q_{i}}=Q_{i}$, equations of motion were calculated for each of the five segment plate masses (see Figure 4), where i is the degree of freedom (displacement for each of the five masses).

**Calculations for the Equation of Motion for mass 1:**

$$\begin{array}{cc} T & =\frac{1}{2}m_{P}{\dot{x}}_{1}^{2}\\ V & =\frac{1}{2}k_{1}x_{S1}^{2}\\ x_{S1} & =x_{1}-x_{2}\\ V & =\frac{1}{2}k_{1}x_{1}^{2}-k_{1}x_{1}x_{2}+\frac{1}{2}k_{1}x_{2}^{2}\\ \frac{d}{dt}\left(\frac{\partial T}{\partial {\dot{x}}_{1}}\right) & =m_{P}{\ddot{x}}_{1}\\ \frac{\partial V}{\partial x_{1}} & =k_{1}\left(x_{1}-x_{2}\right) \end{array}$$

The generalised forces for the equation are ${-SA}_{1}\left[{Fr}_{S}\right]-SB1\left[Fr_{P1}\right]$ which gives the Equation of Motion for mass 1 as:

(A8) $$m_{1}{\ddot{x}}_{1}+k_{1}\left(x_{1}-x_{2}\right)={-SA}_{1}\left[{Fr}_{S}\right]-SB1\left[Fr_{P1}\right]$$

**Calculations for the Equation of Motion for mass 2:**

$$\begin{array}{cc} T & =\frac{1}{2}m_{P}{\dot{x}}_{2}^{2}\\ T & =\frac{1}{2}m_{P}{\dot{x}}_{2}^{2}\\ V & =\frac{1}{2}k_{1}x_{S1}^{2}+\frac{1}{2}k_{2}x_{S2}^{2}\\ V & =\frac{1}{2}k_{1}x_{S1}^{2}+\frac{1}{2}k_{2}x_{S2}^{2}\\ V & =\frac{1}{2}k_{1}x_{S1}^{2}+\frac{1}{2}k_{2}x_{S2}^{2}\\ x_{S1} & =x_{1}-x_{2}\\ x_{S2} & =x_{2}-x_{3}\\ V & =\frac{1}{2}k_{1}x_{1}^{2}-k_{1}x_{1}x_{2}+\frac{1}{2}k_{1}x_{2}^{2}+\frac{1}{2}k_{2}x_{2}^{2}-k_{2}x_{2}x_{3}+\frac{1}{2}k_{2}x_{3}^{2}\\ \frac{d}{dt}\left(\frac{\partial T}{\partial {\dot{x}}_{1}}\right) & =m_{P}{\ddot{x}}_{2}\\ \frac{\partial V}{\partial x_{2}} & =-k_{1}x_{1}+k_{1}x_{2}+k_{2}x_{2}-k_{2}x_{3} \end{array}$$

The generalised forces for the equation are ${SA}_{1}\left[{Fr}_{S}\right]-SA2\left[{Fr}_{S}\right]-SB2\left[Fr_{P2}\right]$ which gives the Equation of Motion for mass 2 as:

(A9) $$m_{P}{\ddot{x}}_{2}-k_{1}x_{1}+k_{1}x_{2}+k_{2}x_{2}-k_{2}x_{3}=-{SA}_{1}\left[{Fr}_{S}\right]-SA2\left[{Fr}_{S}\right]-SB2\left[Fr_{P2}\right]$$

**Calculations for the Equation of Motion for mass 3:**

$$\begin{array}{cc} T & =\frac{1}{2}m_{P}{\dot{x}}_{3}^{2}\\ V & =\frac{1}{2}k_{2}x_{S2}^{2}+\frac{1}{2}k_{3}x_{S3}^{2}\\ x_{S2} & =x_{2}-x_{3}\\ x_{S3} & =x_{3}-x_{4}\\ V & =\frac{1}{2}k_{2}x_{2}^{2}-k_{2}x_{2}x_{3}+\frac{1}{2}k_{2}x_{3}^{2}+\frac{1}{2}k_{3}x_{3}^{2}-k_{3}x_{3}x_{4}+\frac{1}{2}k_{3}x_{4}^{2}\\ \frac{d}{dt}\left(\frac{\partial T}{\partial {\dot{x}}_{3}}\right) & =m_{P}{\ddot{x}}_{3}\\ \frac{\partial V}{\partial x_{3}} & =-k_{2}x_{2}+k_{2}x_{3}+k_{3}x_{3}-k_{3}x_{4} \end{array}$$

The generalised forces for the equation are $-SA_{2}\left[{Fr}_{S}\right]-SA_{3}\left[Fr_{S}\right]-SB_{3}\left[Fr_{P3}\right]$ which gives the Equation of Motion for mass 3 as:

(A10) $$m_{P}{\ddot{x}}_{3}-k_{2}x_{2}+k_{2}x_{3}+k_{3}x_{3}-k_{3}x_{4}=-{SA}_{2}\left[{Fr}_{S}\right]-SA_{3}\left[Fr_{S}\right]-SB3\left[Fr_{P3}\right]$$

**Calculations for the Equation of Motion for mass 4:**

$$\begin{array}{cc} T & =\frac{1}{2}m_{P}{\dot{x}}_{4}^{2}\\ V & =\frac{1}{2}k_{S3}x_{S3}^{2}+\frac{1}{2}k_{S4}x_{S4}^{2}\\ x_{S3} & =x_{3}-x_{4}\\ x_{S4} & =x_{4}-x_{5}\\ V & =\frac{1}{2}k_{3}x_{3}^{2}-k_{3}x_{3}x_{4}+\frac{1}{2}k_{3}x_{4}^{2}+\frac{1}{2}k_{4}x_{4}^{2}-k_{4}x_{4}x_{5}+\frac{1}{2}k_{4}x_{5}^{2}\\ \frac{d}{dt}\left(\frac{\partial T}{\partial {\dot{x}}_{4}}\right) & =m_{P}{\ddot{x}}_{4}\\ \frac{\partial V}{\partial x_{4}} & =-k_{3}x_{3}+k_{3}x_{4}+k_{4}x_{4}-k_{4}x_{5} \end{array}$$

The generalised forces for the equation are $-{SA}_{3}\left[{Fr}_{S}\right]-SA4\left[Fr_{S}\right]-SB_{4}\left[Fr_{P4}\right]$ which gives the Equation of Motion for mass 4 as:

(A11) $$m_{P}{\ddot{x}}_{4}-k_{3}x_{3}+k_{3}x_{4}+k_{4}x_{4}-k_{4}x_{5}=-{SA}_{3}\left[{Fr}_{S}\right]-SA4\left[Fr_{S}\right]-SB_{4}\left[Fr_{P4}\right]$$

**Calculations for the Equation of Motion for mass 5:**

$$\begin{array}{cc} T & =\frac{1}{2}m_{P}{\dot{x}}_{5}^{2}\\ V & =\frac{1}{2}kx_{S4}^{2}\\ x_{S4} & =x_{5}-x_{4}\\ V & ={\frac{1}{2}k}_{4}x_{4}^{2}-k_{4}x_{4}x_{5}+\frac{1}{2}k_{4}x_{5}^{2}\\ \frac{d}{dt}\left(\frac{\partial T}{\partial {\dot{x}}_{5}}\right) & =m_{P}{\ddot{x}}_{5}\\ \frac{\partial V}{\partial x_{5}} & =-k_{4}x_{4}+k_{4}x_{5} \end{array}$$

The generalised forces for the equation are ${SA}_{4}\left[{Fr}_{S}\right]-SB_{4}\left[Fr_{P5}\right]$ which gives the Equation of Motion for mass 5 as:

(A12) $$m_{P}{\ddot{x}}_{5}-k_{4}x_{4}+k_{4}x_{5}=\;-{SA}_{4}\left[{Fr}_{S}\right]-SB_{4}\left[Fr_{P5}\right]$$

## Appendix C. Materials and Methods

### Appendix C.1. Iterations of the Manufacturing Process

**First Trial**

In order to manufacture the 12 PAMs, the first step in the process was developing a CAD model for the mould. The CAD mould for the mould was created using the existing model of the PAM, which had been tested and optimised using FEA. The CAD model for the mould was made from three parts—one inner and two outer moulds. In order to create the mould from the original PAM model, a box was extruded through the model, enclosing the PAM. A Boolean subtract function was then used to hollow out the shape of the PAM from the box. The box was then halved using a split function, and small edits were made to the mould such as adding breathing and pouring holes, filleting, and cutting out unnecessary volumes from the mould to minimise waste. The CAD for the PAM was then amended to create the inner mould. The thickening function was used to thin the PAM, leaving a 1 mm gap between the outer and inner moulds for the material to be poured into. Supports were also added to keep the inner mould stable during the casting process. The initial CAD of the inner and outer moulds is shown in Figure A6.

The inner and outer inner moulds were then 3D-printed in PLA. Multiple printers were tested: a Prusa Mini with a 0.1 mm layer height; an Ultimaker UM3 with a 0.1 mm layer height and a Ultimaker UM3 Fine Detail with a 0.4 mm layer height.

Once the moulds were printed, the silicone was mixed for casting (silicone and curing agent provided, mixed in a 1:1 ratio as intended). To remove air bubbles from the mixture (that may impair the PAM’s surface once set), multiple degassing techniques were tested: vacuum sealer and vibration table, with most success using the table. A vacuum chamber was unfortunately inaccessible.

The moulds were then lubricated with a water-based lubricant, and the inner mould was glued onto one of the outer moulds to keep it in place. Glue was then used to join the two outer moulds together, and then the silicone was poured into the mould. Immediately, the pouring hole was plugged with blue tack, tape, and rubber bands. Vibration was used to minimise the air bubbles as soon as moulds were cast, and the silicone was left to cure for 24 h.

After 24 h, the outer moulds were removed. Once it was confirmed that the silicone could withstand the necessary temperature, the inner mould was then melted from the silicone mould. To ensure that the silicone would not be damaged, a test strip of cured silicon was heated alongside.

From the initial manufacturing trial, there were multiple significant findings:

- The best method for casting the silicone was found to be sealing mould parts together and then pouring silicon, rather than trying to fill moulds with silicon first.
- The optimum temperature for melting the PLA was found to be 170 °C. A test strip of cured silicon was trailed to ensure that this temperature would not alter the properties, and this was the lowest temperature at which the PLA easily melted.
- The Prusa Mini with a 0.1 mm layer height was found to be the most effective printing method in order to maximise the resolution of the print and avoid any gaps in the print which the silicone could sink into during moulding.

**Second Trial**

Following the first trial of manufacturing the PAMs, numerous improvements were made to the PAM design and the manufacturing process:

1. Firstly, the thickness of the membrane of the PAM was increased from 1 mm to 3 mm. This made the PAMs more durable and reduced the precision required in the moulding process.
2. The thickness of the walls was also reinforcing around the holes at the top of the PAM, as this was found by FEA to be a weak point of the design due to increased strain from the fitting.
3. The height and indent of the pouring hole were increased in order to make the casting process easier, and it was decided that a syringe would be used for injection moulding rather than pouring. This would allow the silicon to slowly fill the mould from the bottom up, reducing bubbles and improving the evenness of the coat.
4. The amount of material used in the inner mould was reduced by reducing the infill in the print and making the CAD model less dense in order to make the melting process of the PLA easier.
5. The print was optimised to decrease the print times by reducing the infill and supports to the outer mould where possible.
6. Extra supports for printing were added to the inner mould to improve the reliability of print.
7. A ‘stand’ was added to replace the stems in the original CAD model, as shown in Figure A7, created from cylinder with rivets
8. The holes at the top and the bottom of the mould were enlarged in order to fit the 3.9 valve fittings and 4 mm plugs, as well as increasing the length at the bottom to make space for any clips around the fittings. Having larger holes also makes the casting process easier.

With the following changes made to the manufacturing process, the mould was printed on a Prusa Mini with a 0.1 mm later height. The second design is shown below in Figure A7:

Before injection, the moulds were sprayed with mould release spray. The stand was then glued into the bottom of the mould to prevent it from twisting, using a soft PVA glue. Hot glue was then used to join the outer moulds together, and to glue the inner mould into fixture at top. A syringe was then used to push silicon into mould, and immediately after, the pouring hole was plugged with blue tack, tape, and rubber bands. The mould was then secured onto a vibrating table to remove bubbles for a several hours, and then left to cure for 24 h.

From the second manufacturing trial, there were multiple significant findings:

- The stems were a weak point in the model, with multiple stems snapping before the moulding process.
- The inner mould was able to rotate too much within the outer mould, making for an uneven coating of silicone.
- Injection moulding was not useful because, as the silicone moved up, it sheared on the edges of the mould, causing bubbles which is why this method was not used in the final manufacturing process.

**Final Manufacturing Process**

Following on from the second trial of manufacturing the PAMs, numerous improvements were made to the PAM design and the manufacturing process:

- The design of the stems was changed to make them thicker, eliminating the weak point.
- The stand was removed for the mould design, replaced with a top and bottom fixture which the inner mould could be glued to.
- The PAM’s shape was changed—it was designed using geometrical functions and lofted following the curves which allowed both the inner and outer surfaces to be lofted independently. This allowed the PAM to have the exact thickness built into it.
- Due to the built-in thickness, the subtract function automatically divided the extruded mould box into two parts; one for the inner mould and one for the outer mould. The thicken function was no longer necessary, as these parts were already the correct size and the space in between them would be the empty space to be filled with silicone rubber.
- Small touch-ups were also unnecessary for the new moulds, as the derived PAM part had every detail already incorporated in it, including all the holes (which would become the inner mould “stalks”).
- The mould was instead split along the horizontal plane, to keep the inner mould stable and centred and to make the casting process easier.
- The bottom of the mould was sealed and a small extrusion at the bottom was added to allow the inner mould to “click” into place. Here, a vital correction to the previous mould design was to add 0.4 mm of clearance, as this was a design weakness in the second version.
- A chunk of the outside of the outer mould was removed to drastically reduce the print volume and thus the print time.

### Appendix C.3. Finite Element Analysis (FEA) of External Loads

The head and tail of the worm are designed symmetrically and act as protection for the main body of the PALAM robot. As such, the FEA (finite element analysis) analysis was conducted to determine whether a sudden impact of approximately 100 N would cause material failure in the end plates. Fixed boundary conditions were placed on the outside faces to determine the effect of a direct central impact. In Figure A11, the outcome of a straight-on impact force can be seen. The maximum stress experienced is approximately 1.163 MPA, which is well below the elastic limit of PLA. The minimum strains experienced throughout as shown in Figure 11 of $3.517\times 10^{-5}$ m/m. The stress is well distributed and any sudden increase of up to 100 N would be absorbed.

In Figure A9, the result of an impact to the side of the head/tail is observed. The maximum stress and strain experienced are 3.066 MPa and 7.717 m/m, respectively. Both are observed to be higher than the maximum stress and strain because of a “head-on” impact, because the side is less capable of absorbing impacts. However, the maximum stress values are still way below the elastic limit of PLA, and hence, it can be concluded that the design of the end plates of the PALAM worm is adequate for protection of sudden impacts of up to 100N.

## Appendix D. Discussion

### Appendix D.1. PAM: Manufacturing Results

**Table A3:** Average PAM weight.

PAM No.	Weight (g)
1	16
2	16
3	18
4	17
5	18
6	17
7	18
8	16
9	17
10	17
11	17
12	17

### Appendix D.2. PAM Testing

The time taken for the maximum inflation of a single PAM was measured 10 times, as shown in Table A4. The average time for inflation is 1.06 s.

**Table A4:** Inflation time (s) for a single PAM.

Measure No.	Time Taken (s)
1	0.81
2	0.76
3	0.80
4	0.79
5	0.83
6	0.78
7	0.80
8	0.82
9	0.78
10	0.81

The inflation times of the PAMs in the full assembly were then measured, as shown in Table A5.

**Table A5:** Inflation time (s) for multiple PAMs.

Measure No.	Time Taken (s)
1	1.05
2	1.04
3	1.09
4	1.07
5	1.05
6	1.08
7	1.07
8	1.07
9	1.05
10	1.06
